# Gated Transient Dissipative Dimerization of DNA Tetrahedra Nanostructures for Programmed DNAzymes Catalysis

**DOI:** 10.1021/acsnano.1c06117

**Published:** 2022-02-20

**Authors:** Zhenzhen Li, Jianbang Wang, Zhixin Zhou, Michael P. O’Hagan, Itamar Willner

**Affiliations:** The Institute of Chemistry, The Center for Nanoscience and Nanotechnology, The Hebrew University of Jerusalem, Jerusalem 91904, Israel

**Keywords:** DNA nanotechnology, protein−protein interactions, Mg^2+^-ion-dependent DNAzyme, nicking enzyme, dynamic network

## Abstract

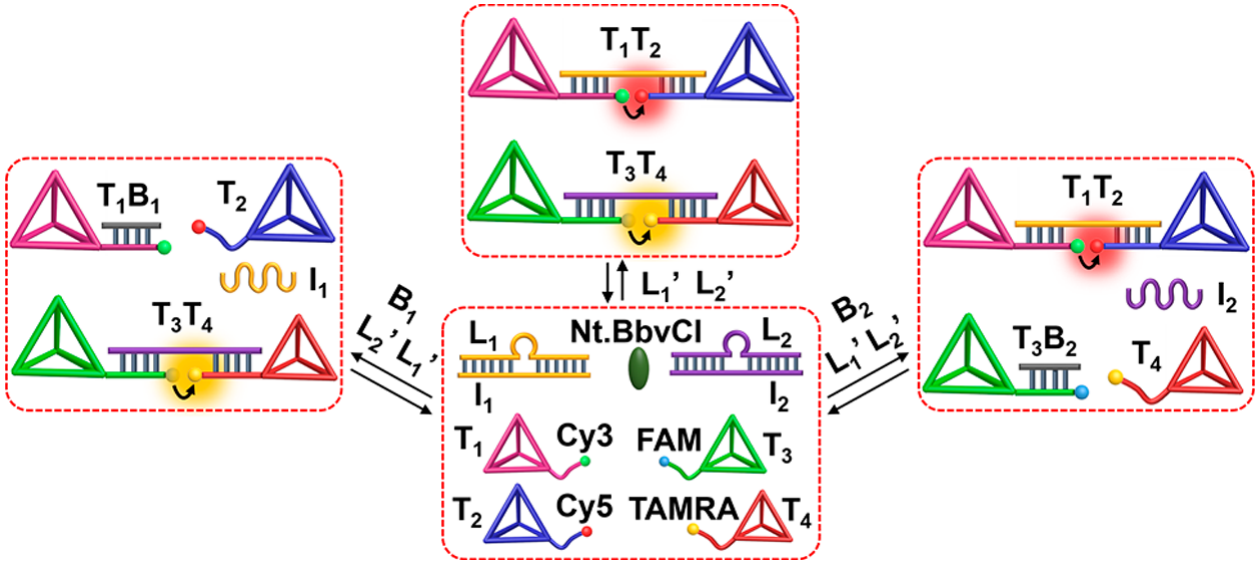

Transient dissipative dimerization and transient gated dimerization of DNA tetrahedra nanostructures are introduced as functional modules to emulate transient and gated protein–protein interactions and emergent protein–protein guided transient catalytic functions, operating in nature. Four tetrahedra are engineered to yield functional modules that, in the presence of pre-engineered auxiliary nucleic acids and the nicking enzyme Nt.BbvCI, lead to the fueled transient dimerization of two pairs of tetrahedra. The dynamic transient formation and depletion of DNA tetrahedra are followed by transient FRET signals generated by fluorophore-labeled tetrahedra. The integration of two inhibitors within the mixture of the four tetrahedra and two auxiliary modules, fueling the transient dimerization, results in selective inhibitor-guided gated transient dimerization of two different DNA tetrahedra dimers. Kinetic models for the dynamic transient dimerization and gated transient dimerization of the DNA tetrahedra are formulated and computationally simulated. The derived rate-constants allow the prediction and subsequent experimental validation of the performance of the systems under different auxiliary conditions. In addition, by appropriate modification of the four tetrahedra structures, the triggered gated emergence of selective transient catalytic functions driven by the two pairs of DNA tetrahedra dimers is demonstrated.

Transient formation and dissociation of protein–protein complexes represent key processes in nature orchestrating signaling transductions, electron transport chains, and activations of dynamic networks and operating biocatalytic cascades and branched biocatalytic cycles.^1−5^ Bioprocesses such as transcriptional regulation of cellular activities,^6^ proliferation and differentiation of cells,^7,8^ or cell–cell recognition and adhesion^9,10^ are regulated by transient protein–protein interactions. Emulating transient natural protein–protein functionalities by synthetic constituents is a major challenge in the rapidly developing area of Systems Chemistry.^11−13^ DNA tetrahedra nanostructures attract recent research interest as a functional material for various applications.^14−17^ The stability of DNA tetrahedra, their size-tunability by the lengths of the comprising nucleic acid strands, and the ease of functionalization of the corners or edges of the tetrahedra structure, turn these nanostructures as ideal components for various uses.^18,19^ Indeed, different applications of DNA tetrahedra were suggested, including their use for sensing and multiplex sensing,^20−22^ intracellular imaging,^23−25^ nanocarriers of loads,^26,27^ and nanoscale scaffolds for engineering chiroplasmonic nanostructures.^28^ The sizes of DNA tetrahedra, 3–12 nm, and their superior cell permeation properties^26,29,30^ introduce objects of dimensions and cell permeability features comparable to proteins, and thus, by appropriate functionalization of their structures, DNA tetrahedra could act as functional modules mimicking protein–protein interactions. Furthermore, similar to embed catalytic sites in proteins, one could introduce a catalytic site into the tetrahedra scaffolds with distinct variable catalytic functions of “inner” or “outer” catalysts positioned in the tetrahedra structures^31^ (for further discussion on these structural and functional properties, *vide infra*). Indeed, a recent report demonstrated the triggered, thermodynamically controlled, dynamic reconfiguration of constitutional dynamic networks consisting of DNA tetrahedra, as a model for protein–protein interactions.^19^ Transient, out-of-equilibrium, dynamic operations of DNA tetrahedra, as a means to emulate transient protein–protein interactions are desirable. Particularly, the design of gated transient transitions of DNA tetrahedra, and the development of transient DNA-tetrahedra-guided gated catalytic transformations are important to model transient protein–protein interactions. It should be noted that the DNA tetrahedra scaffolds are very distant in their structural and functional complexities compared to proteins. The size resemblance of DNA tetrahedra to small-sized proteins and the modularity to tether structural or catalytic strands to the DNA tetrahedra and their cell permeability and carrier properties, however, provide biomimetic Systems Chemistry tools to model proteins.

The design of artificial out-of-equilibrium systems attracts substantial recent research efforts. Examples include the biocatalytic amination and hydrolysis of peptides leading to spatiotemporal assembly and disassembly of fibers,^32^ the transient assembly of vesicles through noncovalent interactions between surfactants and adenosine triphosphate (ATP), acting as fuel, and their separation upon hydrolysis of ATP.^33^ Also, the guanosine triphosphate transient assembly of the FtsZ protein within coacervate droplets into fibrils elongates the coacervate droplets, leading to their separation and the division of the protein fibrils, as a model for cell division,^34^ and the demonstration of dissipative chlorophyll-to-carotenoid energy transfer in the light-harvesting complex II in membrane nanodiscs^35^ represents synthetic transient model systems mimicking biological transformations. It should be noted that some terminological discrepancies defining nonequilibrium systems exist.^36^ In fact, substantial discussions addressed the relation between energy/fuel input into chemical systems and the formation of nonequilibrium, transient products.^37−39^ Accordingly, we consider a dissipative system as a self-assembled module that requires the supply of energy or a chemical fuel to yield waste products and nonequilibrated reaction intermediates undergoing transient transitions to the original self-assembled state.

The information encoded in the base sequence of nucleic acids provides a rich “toolbox” to construct dynamic networks. Thermodynamically controlled equilibrated reconfiguration of constitutional dynamic networks were reported,^40−43^ and their application as functional modules to generate triggered formation of hydrogel materials of controlled stiffness,^44^ to guide transcription and translation processes,^45^ and to intercommunicate biocatalytic cascades^46^ were reported. Also, out-of-equilibrium transcriptional circuits acting as transcriptional oscillators^47,48^ or transcriptional switches and bistable regulatory networks^49^ were demonstrated. Furthermore, enzyme-based DNA machinery relying on polymerization/nickase or sequence-specific nicking enzymes were applied to assemble out-of-equilibrium oscillatory behaviors^50−52^ and transient operation of constitutional dynamic networks.^53^ Also, light-driven ATP-fueled out-of-equilibrium DNA ligation cycles^54,55^ were reported. In the present study, we report on the transient dimerization of DNA tetrahedra and discuss the analogy between the dynamic function of these nanostructures and native protein–protein interactions. Particularly, we introduce means to gate the transient selection of two different dimer pairs from a mixture of tetrahedra nanostructures, as a means to model selective gating of protein–protein interactions within an ensemble of proteins. In addition, we conjugate the gated DNA tetrahedra structures to functional nucleic acid tethers, leading to emerging catalytic functions of the respective dimers, in analogy to guided catalytic functions dictated by protein–protein interactions. The dynamics of the transient and gated transient modules are accompanied by kinetic modeling of the systems using computational simulations. The derived rate constants are applied to predict the transient behaviors of the systems subjected to different auxiliary triggers, and the predicted results are validated by experiments. It should be noted that in a recent study, we reported on the execution of a related, transient system comprising of duplex nucleic acid constituents. The advances of the present systems rest, however, on the dynamic formation of transient and gated transient dimeric protein-mimetic tetrahedra nanostructures and demonstrate emerging protein/protein-like catalytic functions. This would allow the integration of such systems into protocell assemblies.

## Results and Discussion

**Figure 1:**
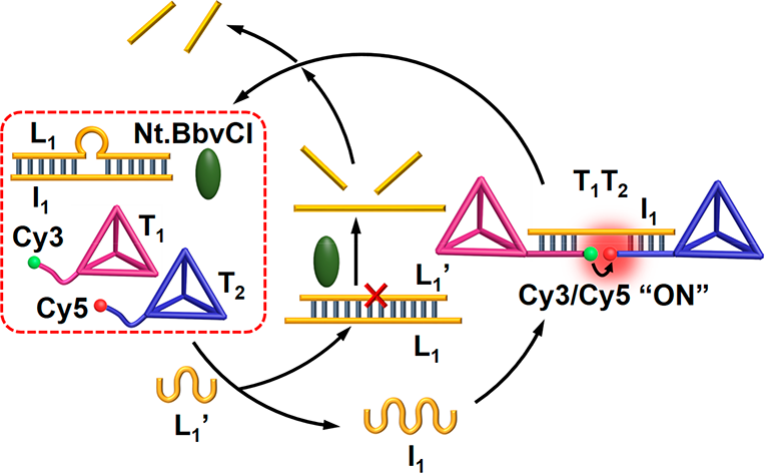
Scheme for the transient formation and depletion of the T_1_/T_2_ dimer tetrahedra nanostructure. The dissipative process is followed by the transient FRET signal generated upon formation and depletion of the tetrahedra dimer structures.

**Figure 2:**
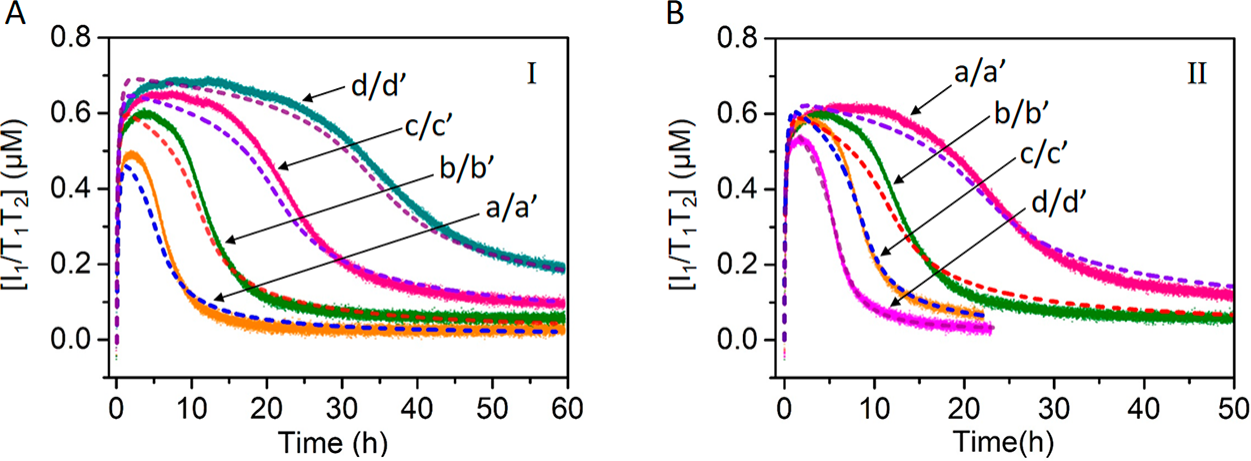
(A) Transient concentration changes of the tetrahedra T_1_/T_2_ upon the L_1_′ fueled, triggered formation and depletion of the T_1_/T_2_ dimer in the presence of variable concentrations of the fuel strand L_1_′ = (a) 2, (b) 4, (c) 6, (d) 8 μM. For all systems: L_1_/I_1_ = 1 μM; T_1_ = 1 μM; T_2_ = 1 μM; and Nt.BbvCI = 0.046 μM (Solid lines correspond to experimental results. Dashed curves a′, b′, c′, and d′ correspond to the computationally simulated transients, using the kinetic model presented in the Supporting Information, Figure S4). Curves b/b′ correspond to the experimental transient (solid line) followed by the computational simulation (dash line), using the kinetic model, leading to the derived rate constants tabulated in Table S1. Curves a/a′, c/c′, and d/d′ were first computationally simulated and subsequently experimentally validated. (B) Transient concentration changes of the tetrahedra T_1_/T_2_ upon the L_1_′ fueled, triggered formation and depletion of the T_1_/T_2_ dimer in the presence of variable concentrations of Nt.BbvCI = (a) 0.0306, (b) 0.046, (c) 0.0612, (d) 0.0765 μM. For all systems: L_1_/I_1_ = 1 μM; T_1_= 1 μM; T_2_ = 1 μM; and fuel strand L_1_′ = 4 μM (Solid lines correspond to experimental results. Dashed curves a′, b′, c′, and d′ correspond to the computational simulated transients, using the kinetic model presented in the Supporting Information, Figure S4). Curves b/b′ correspond to the experimental transient (solid line) followed by the computational simulation (dash line), using the kinetic model, leading to the derived rate constants tabulated in Table S1. Curves a/a′, c/c′, and d/d′ were first computationally simulated and subsequently experimentally validated.

Figure 1 depicts the scheme to operate transient dimerization of two tetrahedra T_1_ and T_2_. The reaction module consists of two tetrahedra T_1_ and T_2_ modified with single stranded tethers functionalized with fluorophores Cy3 and Cy5, respectively, the duplex L_1_/I_1_, and the nicking enzyme Nt.BbvCI. In the presence of the fuel strand L_1_′, the duplex L_1_/I_1_ in the module is displaced to yield the energetically stabilized duplex L_1_/L_1_′ and the free strand I_1_. The constituents of the reaction module are pre-engineered such that the released strand I_1_ bridges the free tethers associated with tetrahedra to yield the dimer T_1_/T_2_, while the resulting duplex L_1_/L_1_′ is designed to include in the L_1_′ strand the specific sequence domain to be nicked by the nicking enzyme to yield two fragments, being separated as “waste” products, thus regenerating the single strand L_1_. The released L_1_ displaces the strand I_1_ bridging the dimer units, resulting in the separation of the dimer, and the recovery of the original rest module. That is, subjecting the reaction module to the fuel strand L_1_′ leads to the dynamic transient formation of the bridged dimer T_1_/T_2_ that undergoes a transient recovery to the initial rest state of separated tetrahedra. For a further discussion explaining the energetics of the transient process and the design principles of the system; see Figure S1. The transient assembly and disassembly of the dimer are probed by the Förster resonance energy transfer (FRET) process proceeding between the fluorophores Cy3 and Cy5 in the intimate T_1_/T_2_ dimer structure. Figure S2 depicts the time-dependent fluorescence changes of Cy3 and Cy5 that follow the dynamic transient formation and disappearance of the dimer structure T_1_/T_2_. Using the appropriate calibration curve relating the fluorescence intensities of fluorophores Cy5/Cy3 to variable concentrations of the dimer T_1_/T_2_, *e.g*., I_1_/T_1_T_2_, Figure S3, the transient fluorescence changes observed upon triggered the reaction module with L_1_′, Figure S2, were translated into transient curves corresponding to the time-dependent dynamic concentration changes upon the formation and disappearance of the dimer T_1_/T_2_, and this is displayed in Figure 2, panel I curve b and panel II curve b. The transient assembly of the dimer T_1_/T_2_ was computationally modeled. The kinetic model accounting for the transient formation and depletion of the T_1_/T_2_ dimer was formulated, and the set of rate constants that follow the kinetic steps involved with the formation and the dissipative depletion of the constituent T_1_/T_2_ are summarized in the Supporting Information Figure S4. The computationally simulated curve b′ was fitted to the experimental results, and the derived rate constants, corresponding to the reaction steps involved in the kinetic model, are summarized in Table S1. To support the derived set of the simulated rate constants, it is important to try to evaluate, independently, experimentally, (and computationally) one (or more) of the rate constants involved in the overall kinetic model. Accordingly, we evaluated independently the values *k*_3_ and *k*_–3_, and the results are presented in Figure S5. The derived values *k*_3_ and *k*_–3_ fit well with the respective rate constants in the overall simulated model, supporting the simulation process. Also, the kinetic model and the resulting derived rate constants have scientific meaning only if some of the rate constants can be experimentally validated and if the set of the rate constants can predict the behavior of the system under variable auxiliary conditions and subsequently validated experimentally. The transient assembly and depletion of the dimer T_1_/T_2_ is anticipated to be affected by the concentrations of the fuel strand L_1_′ and by the concentrations of the nicking enzyme Nt.BbvCI. Accordingly, the computed rate-constants were applied to predict the transient behavior of the system at three different concentrations of L_1_′, 2, 6, and 8 μM (curves a′, c′, and d′, dashed lines), Figure 2, panel I, and at three different concentrations of the nicking enzyme (0.0306, 0.0612, and 0.0765 μM, curves a′, c′, and d′, dashed lines), Figure 2, panel II. The computationally simulated results were then experimentally validated to yield the curves a, c, and d, panel I and the curves a, c, and d, panel II. The experimental results fit well with the predicted transients, indicating the significance of the computational model to understand the kinetic behavior of the system under different conditions. The results demonstrate that as the concentrations of the fuel strand increase, the content of intermediate dimer complex increases, and as the concentration of the nicking enzyme increases, the depletion of the dimer T_1_/T_2_ is enhanced. A further independent method to follow the dynamic transient dimerization of T_1_/T_2_ applied time-dependent quantitative electrophoretic separation of the monomer/dimer constituents. For a detailed discussion, see page s9 and Figure S6 in the Supporting Information.

**Figure 3:**
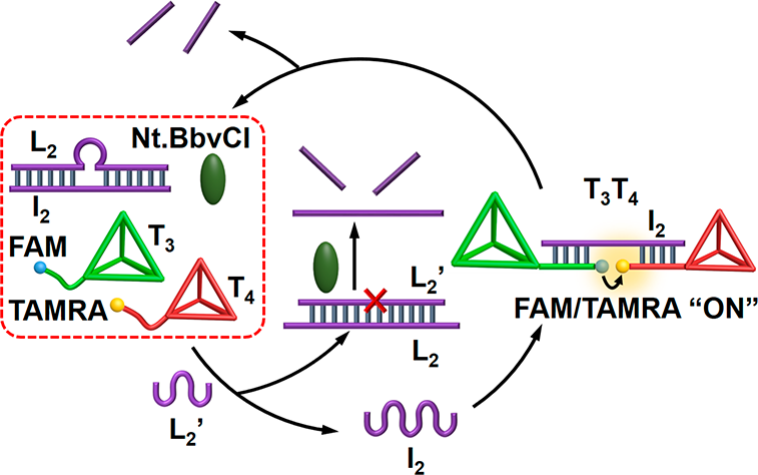
Schematic composition of a FAM-modified T_3_ and TAMRA-functionalized T_4_ tetrahedra mixture in a module that includes the L_2_/I_2_ duplex and the enzyme Nt.BbvCI for the L_2_′-fueled transient dimerization and separation of the T_3_/T_4_ tetrahedra dimer. The transient dimerization is followed by the FRET process between FAM/TAMRA.

Using a similar concept, a second DNA tetrahedra pair of T_3_ and T_4_ was engineered, Figure 3. In this system, the reaction module consists of the FAM-modified T_3_ and the TAMRA-functionalized T_4_, the duplex L_2_/I_2_, and the nicking enzyme Nt.BbvCI. In the presence of the fuel strand L_2_′, the transient formation of the dimer T_3_/T_4_ is activated, followed by the nicking enzyme-stimulated depletion of the dimer structure and the recovery of the initial rest system. The transient formation and the depletion of the intermediate dimer T_3_/T_4_ is followed by the FRET process between the FAM and TAMRA fluorophores. Using an appropriate calibration curve monitoring the FRET signal as a function of different concentrations of the intact T_3_/T_4_ dimer (Figure S7), the time-dependent transient concentration changes of T_3_/T_4_ were evaluated, and the respective transient curve was displayed in Figure S8, curve c, panel I. As before, the transient curve was computationally simulated, curve c′ (see the kinetic model, Figure S9, and derived rate constants, Table S2). The derived rate constants were, then, applied to predict the time-dependent transient curves of the T_3_/T_4_ dimer, in the presence of different concentrations of L_2_′ and the nicking enzyme, Nt.BbvCI, curves a′, b′, and d′, panel I, and curves a′, b′, c′, and d′, panel II, Figure S8, and the results were experimentally validated, curves a, b, and d, panel I, and curves a, b, c, and d, panel II, Figure S8. The experimental results fit well with the time-dependent transient corresponding to T_3_/T_4_, in the presence of the different triggers.

**Figure 4:**
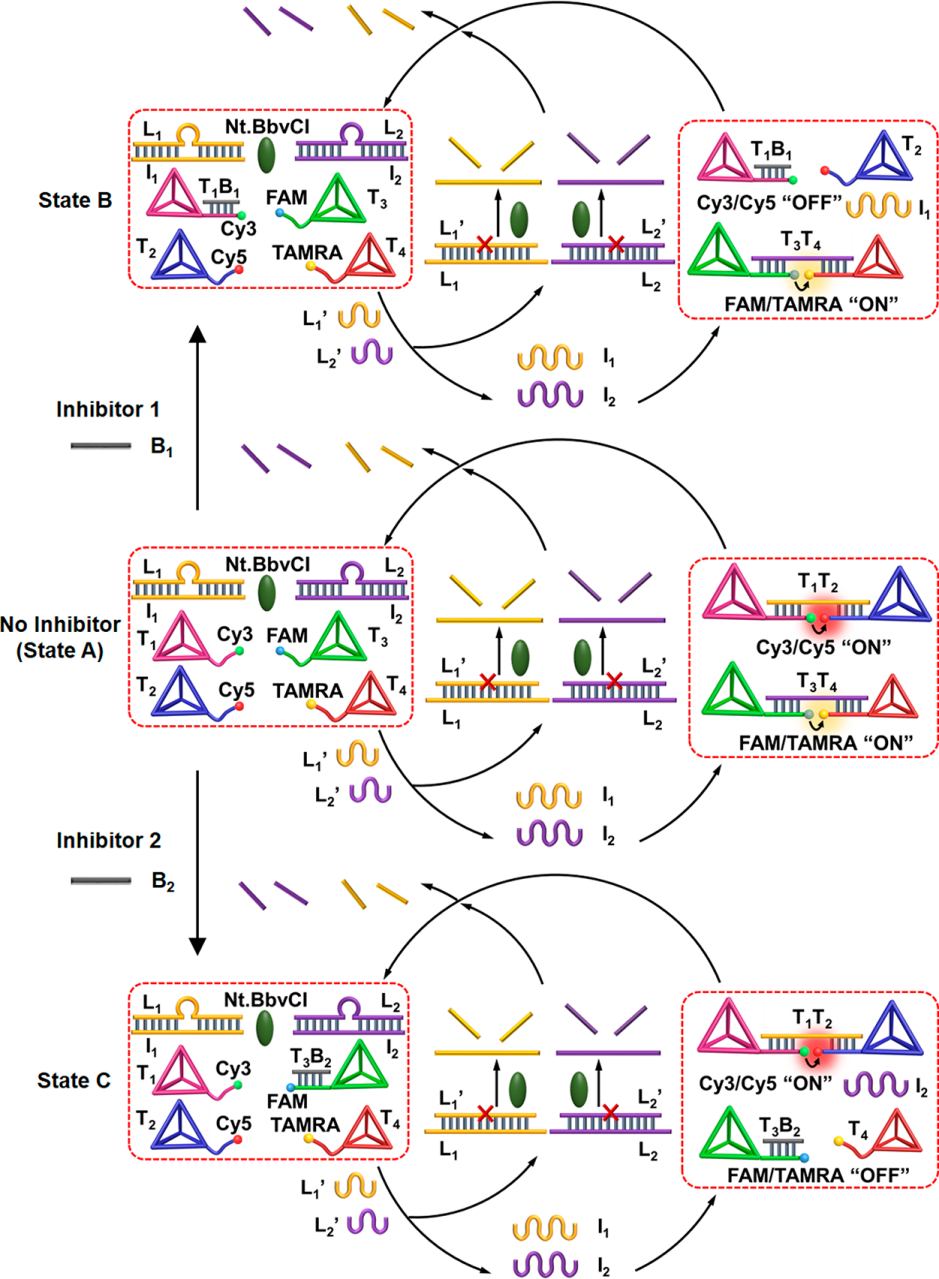
Scheme corresponding to the inhibitor-guided gated transient dimerization of the tetrahedra T_1_/T_2_ and/or T_3_/T_4_. In the absence of the inhibitors, the concomitant dimerization of T_1_/T_2_ and T_3_/T_4_ proceeds. In the presence of inhibitor B_1_, the gated transient dimerization of T_3_/T_4_ occurs; in the presence of B_2_ as inhibitor, the guided gated transient dimerization of T_1_/T_2_ proceeds.

**Figure 5:**
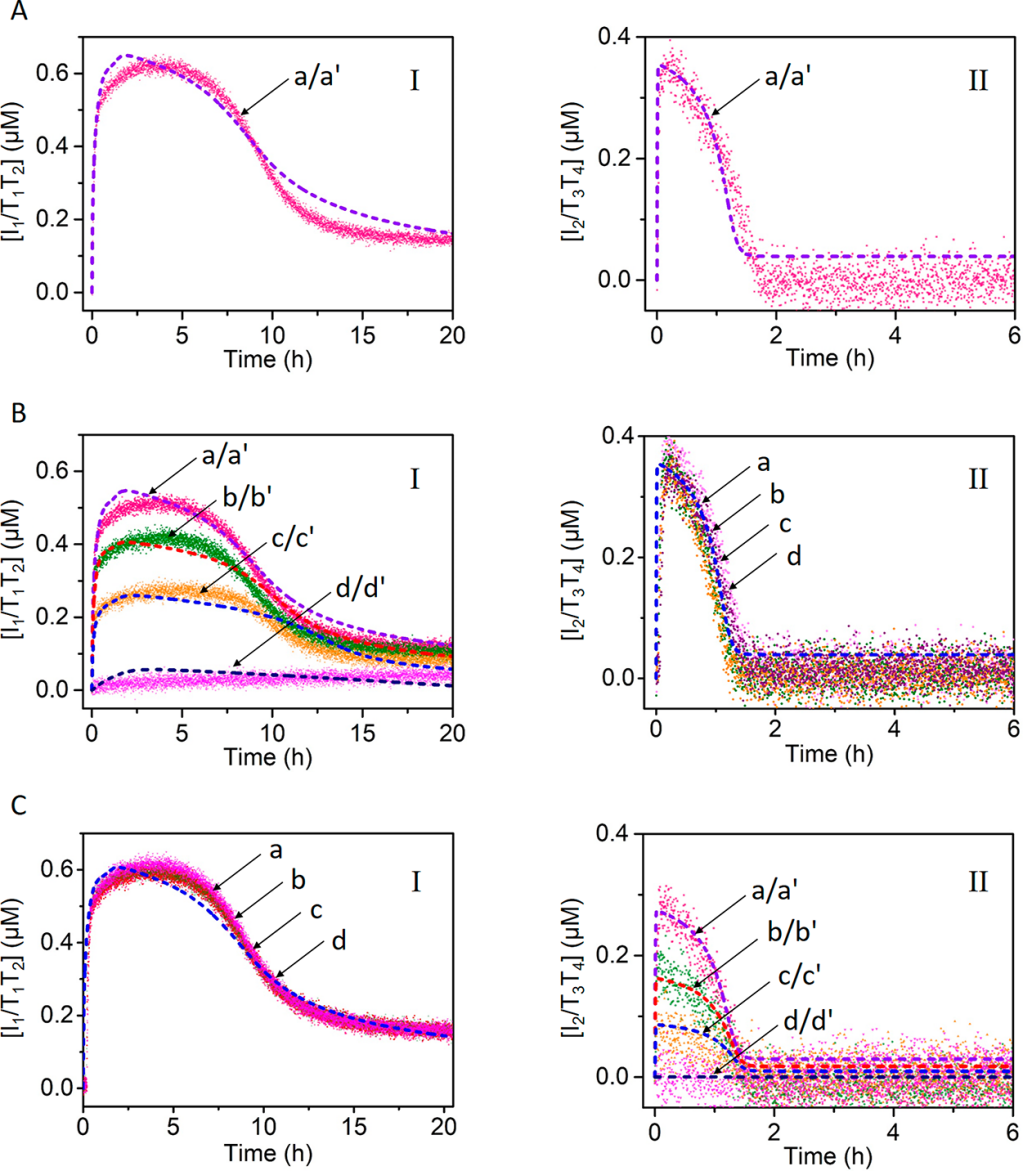
(A) Concomitant transient dimerization of T_1_/T_2_, panel I, curve a, and of T_3_/T_4_, panel II, curve a, upon the L_1_′ and L_2_′ triggered activation of the module in state **A**. The curves a′ in panel I and a′ in panel II correspond to the computationally simulated results (see Supporting Information Figure S11 and Table S3). (B, panel I) Transient dimerization of T_1_/T_2_ in the presence of variable concentrations of B_1_ = (a) 0.33, (b) 0.66, (c) 1, (d) 1.33 μM (a′, b′, c′, and d′ computationally simulated transients). (panel II) Transients of dimer T_3_/T_4_ upon subjecting the module in state **A** to variable concentrations of B_1_ = (a) 0.33, (b) 0.66, (c) 1, (d) 1.33 μM. (C, panel I) Transient dimerization of T_1_/T_2_ in the presence of variable concentrations of B_2_ = (a) 0.33, (b) 0.66, (c) 1, (d) 1.33 μM. (panel II) Transients of dimer T_3_/T_4_ upon subjecting the module in state **A** to variable concentrations of B_2_ = (a) 0.33, (b) 0.66, (c) 1, (d) 1.33 μM (a′, b′, c′, and d′ computationally simulated transients).

The availability of two different tetrahedra dimer structures revealing transient formation and depletion of T_1_/T_2_ and T_3_/T_4_ driven by the fuel strands L_1_′ and L_2_′ and the nicking enzyme suggests that mixing the four tetrahedra T_1_, T_2_, T_3_, and T_4_ could lead to the concomitant transient formation and dissociation of the two dimers T_1_/T_2_, T_3_/T_4_, and to the guided gated formation of T_1_/T_2_ or T_3_/T_4_, in the presence of appropriate inhibitors, as outlined in Figure 4. The mixture of tetrahedra T_1_, T_2_, T_3_, and T_4_ in the presence of the two triggering fuels, L_1_′ and L_2_′, and the nicking enzyme, is anticipated to activate the parallel activation of the transient formation and dissociation of the dimers T_1_/T_2_ and T_3_/T_4_, state **A**. In the presence of inhibitor B_1_ that blocks the free tether associated with T_1_ (forming T_1_B_1_), the triggered formation of T_1_/T_2_ is inhibited, whereas the L_2_′-triggered formation/depletion of T_3_/T_4_ is feasible, state **B**. As the concentration of the inhibitor B_1_ increases, the blockage of the transient formation of T_1_/T_2_ should be enhanced. Similarly, the introduction of the inhibitor B_2_ to the reaction mixture blocks the free tether associated with T_3_ to form T_3_B_2_, resulting in the blockage of the transient formation of T_3_/T_4_, whereas the formation of T_1_/T_2_ proceeds with no interference, state **C**. That is, the mixture of the four tetrahedra could emulate the formation/dissociation of protein–protein interactions, and particularly emulate the inhibition of protein–protein binding interactions guided by auxiliary inhibiting triggers. The gated operation of the transients corresponding to T_1_/T_2_ and T_3_/T_4_, in the presence of the respective inhibitors, is demonstrated in Figure 5. In the absence of the inhibitors, the two transient processes generating T_1_/T_2_ and T_3_/T_4_ proceed, Figure 5A, panels I and II. In the presence of inhibitor B_1_, the transient formation of T_1_/T_2_ is inhibited, Figure 5B, panel I, whereas the transient formation of T_3_/T_4_ is unaffected, Figure 5B, panel II. As the concentration of B_1_ is elevated, the degree of inhibition of the transient formation of T_1_/T_2_ increases. At a concentration of B_1_ corresponding to 1.33 μM, the formation of T_1_/T_2_ is fully blocked. The effect of the inhibitor B_1_ on the transient formation and depletion of T_1_/T_2_ were kinetically modeled (see Supporting Information Figures S11–S13). The derived rate constants are tabulated in Tables S3–S5. The computationally simulated transient curves are presented in curves a′, b′, c′, and d′, Figure 5B, panel I (In fact, the experimental curve b was simulated to yield b′ and the derived rate-constants were used to predict the transients at different concentrations of B_1_, and the computational results were subsequently validated by experiments). Very good fit between the experiments and computationally simulated results is observed. Similarly, the gated operation of the transient formation of T_3_/T_4_, in the presence of the inhibitor B_2_ is displayed in Figure 5C, panels I and II. In the presence of B_2_ the formation and depletion of T_3_/T_4_ is inhibited, Figure 5C, panel II. The degree of inhibition is controlled by the concentrations of B_2_, and as the concentration of B_2_ increases, the blockage of T_3_/T_4_ is higher, and at a concentration of B_2_, corresponding to 1.33 μM, the process generating T_3_/T_4_ is fully blocked. At the same time the transient formation of T_1_/T_2_ is unaffected upon the addition of B_2_. As before, the effect of added B_2_ on the transients generating T_3_/T_4_ was kinetically modeled, and the fit of the computationally simulated transients, curves a′, b′, c′, and d′ are validated by experiments (curves a, b, c, and d in Figure 5C, panel II).

**Figure 6:**
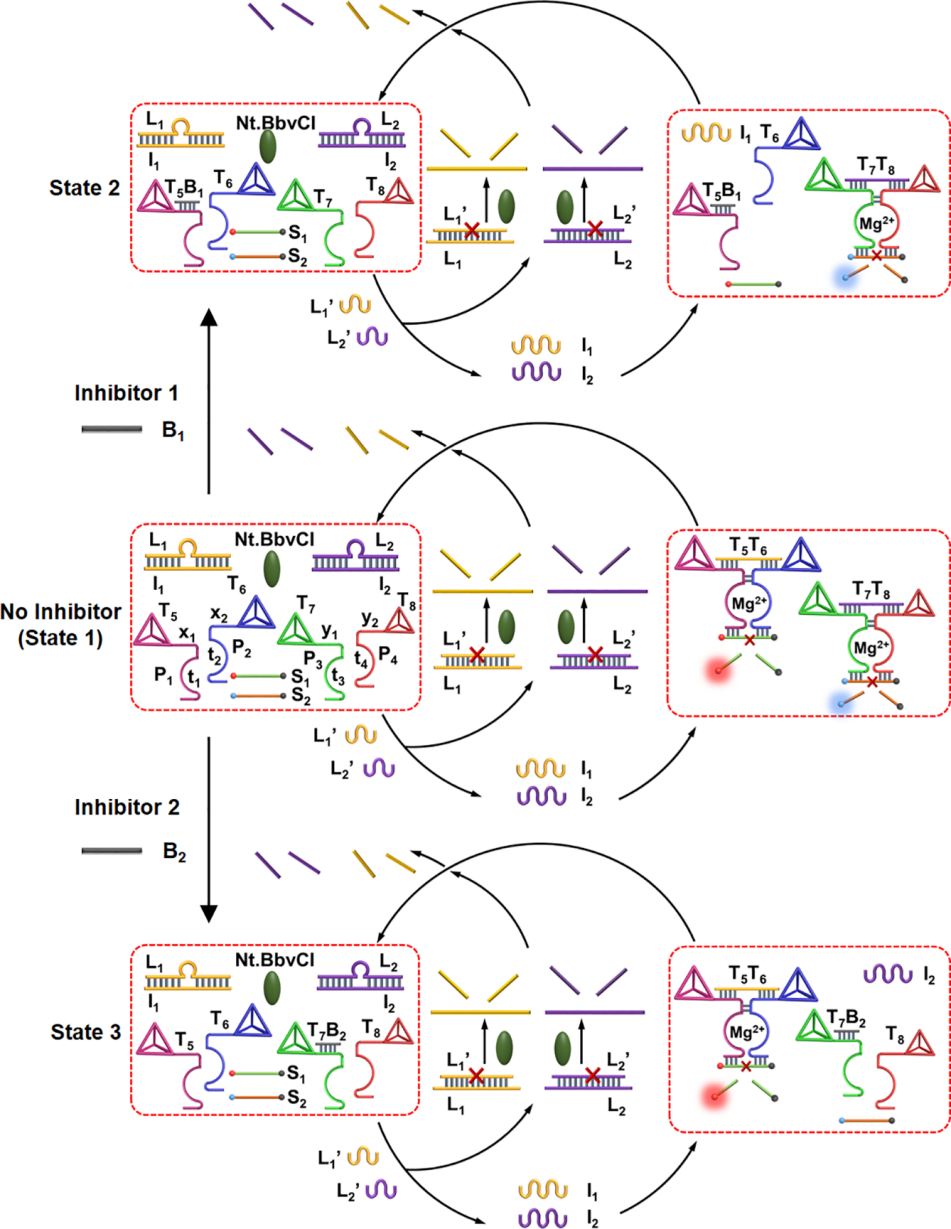
Scheme corresponding to the inhibitor-guided gated dynamic formation of transient DNAzymes catalytic formations. State **1**: In the absence of the inhibitors, the two DNAzymes, DNAzyme(**1**) and DNAzyme(**2**), are formed as transient catalytic outputs. State **2**: In the presence of inhibitor B_1_, only the transient DNAzyme(**2**) is formed. State **3**: In the presence of inhibitor B_2_, only the transient DNAzyme(**1**) is operative.

**Figure 7:**
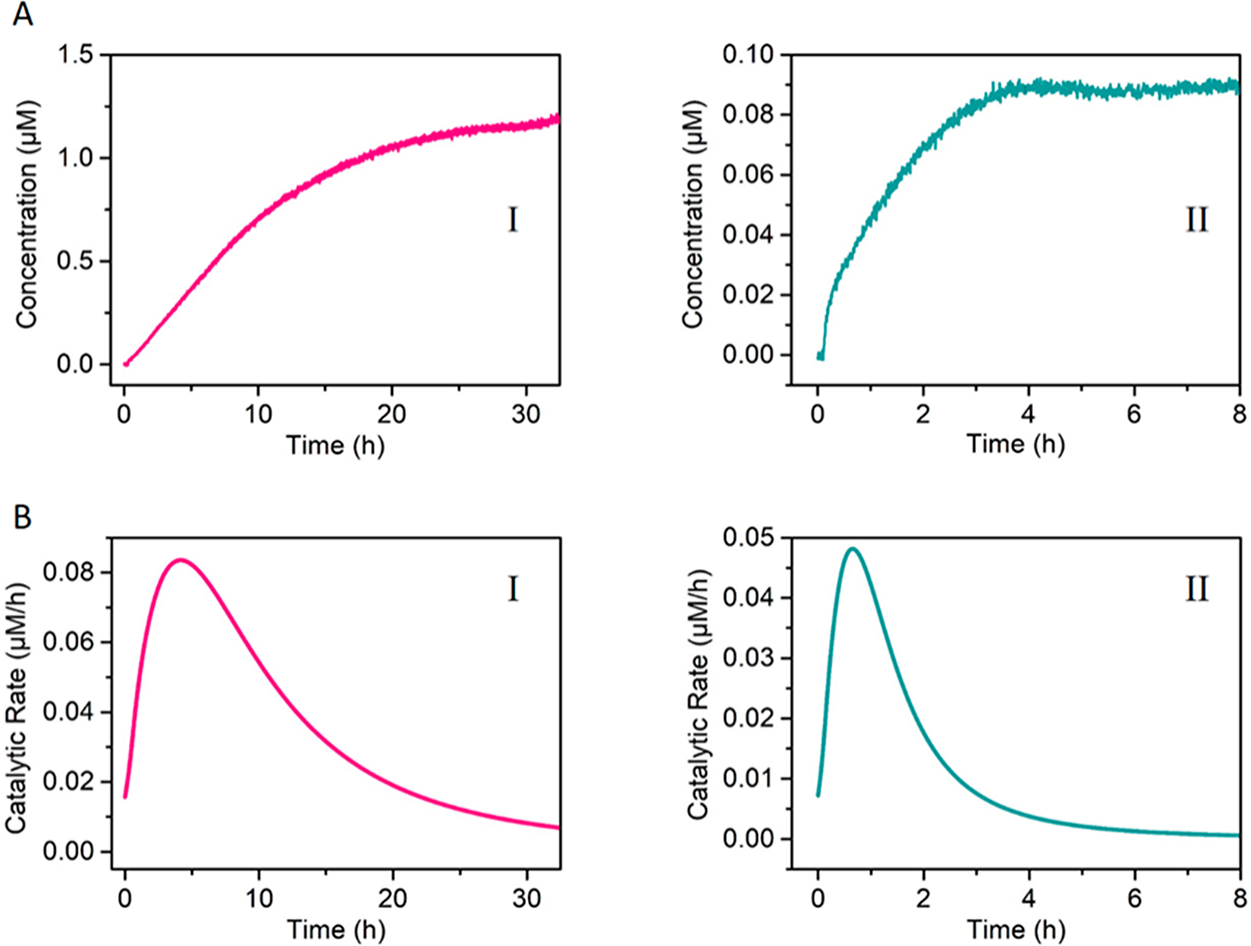
Following the time-dependent catalytic rates of DNAzyme(**1**) and DNAzyme(**2**) in state **1**, Figure 6. (A) Time-dependent concentration changes of (panel I) the Cy5-labeled fragmented product of DNAzyme(**1**); (panel II) the FAM-labeled fragmented product of DNAzyme(**2**). (B) Transient catalytic rates corresponding to (panel I) DNAzyme(**1**); (panel II) DNAzyme(**2**).

Finally, the gated operation of the transient formation and dissociation of the tetrahedra dimers T_1_/T_2_ and T_3_/T_4_ were applied to demonstrate emerging catalytic processes guided by the respective dimer structures. That is, in analogy to natural processes where protein–protein interactions lead to emerging catalytic functions, the dimer tetrahedra nanostructures emulate the processes in nature. Figure 6 depicts the gated transient catalytic functions guided by the tetrahedra dimer structures. Four different tetrahedron structures T_5_, T_6_, T_7_, and T_8_ were designed. The tetrahedra T_5_ and T_6_ include tethers P_1_ and P_2_ composed of sequence domains x_1_ and x_2_ that are extended by DNAzyme subunits t_1_ and t_2_ that can assemble under appropriate conditions to the Mg^2+^-ion-dependent DNAzyme(**1**) that cleaves the fluorophore/quencher-functionalized substrate S_1_ (fluorophore = Cy5, quencher = BHQ2). The sequence domain x_1_ and x_2_ are complementary to I_1_ being part of the L_1_/I_1_ module. Similarly, the tetrahedra T_7_ and T_8_ are functionalized with the tethers P_3_ and P_4_ that are composed of the sequence domains y_1_ and y_2_ extended by the DNAzyme subunits t_3_ and t_4_, respectively. The domains y_1_ and y_2_ are complementary to the strand I_2_ associated with the module L_2_/I_2_, and the subunits t_3_ and t_4_ assemble under appropriate conditions to the Mg^2+^-ion-dependent DNAzyme(**2**) that cleaves the fluorophore/quencher-functionalized substrate S_2_ (fluorophore = FAM, quencher = IBRQ). The reaction unit in state **1** includes two modules that are triggered in the presence of the fuel strands L_1_′ and L_2_′, to generate two parallel transient catalytic processes driven by DNAzyme(**1**) and DNAzyme(**2**). That is, the fuel strands L_1_′ and L_2_′ separate the modules L_1_/I_1_ and L_2_/I_2_ to yield L_1_/L_1_′ and L_2_/L_2_′, while releasing the strands I_1_ and I_2_. The released strands I_1_ and I_2_ bridge the tetrahedra pairs T_5_, T_6_ and T_7_, T_8_ to yield the dimer tetrahedra T_5_/T_6_ and T_7_/T_8_ that stabilize the respective DNAzyme(**1**) and DNAzyme(**2**) subunits and form the Mg^2+^-ion-dependent DNAzyme(**1**) and DNAzyme(**2**) that cleave the respective substrates S_1_ and S_2_. Cleavage of the substrates releases the respective fluorophore-modified fragmented products that transduce the catalytic functions of DNAzyme(**1**) and DNAzyme(**2**). The L_1_/L_1_′ and L_2_/L_2_′ duplexes are nicked, however, by the nicking enzyme Nt.BbvCI resulting in the release of L_1_ and L_2_, respectively. The released L_1_ and L_2_ strands displace the bridging units I_1_ and I_2_ to form the energetically stabilized original duplexes L_1_/I_1_ and L_2_/I_2_, while separating the dimer tetrahedra T_5_/T_6_ and T_7_/T_8_. The separation of the tetrahedra leads to the separation of DNAzyme(**1**) and DNAzyme(**2**), resulting in the transient depletion of the catalytic functions of the system. The dynamic formation of the catalytic units and their depletion are, then, followed by the time-dependent fluorescence changes of the fluorophores Cy5 and FAM, respectively. Figure S14, panels I and II, shows the transient fluorescence intensities of the fluorophores Cy5 and FAM, upon operation of the catalytic mixture of state **1** in Figure 6. Using the appropriate calibration curves of Cy5-labeled and FAM-modified fragmented products generated by DNAzyme(**1**) and DNAzyme(**2**), Figures S15–S16, the transient concentrations of the fragmented products were evaluated, Figure 7A, panels I and II, and the transient catalytic rates of DNAzyme(**1**) and DNAzyme(**2**) were derived (first-order derivatives of the time-dependent concentrations depicted in panels I and II, Figure 7A), and these are displayed in Figure 7B, panels I and II.

**Figure 8:**
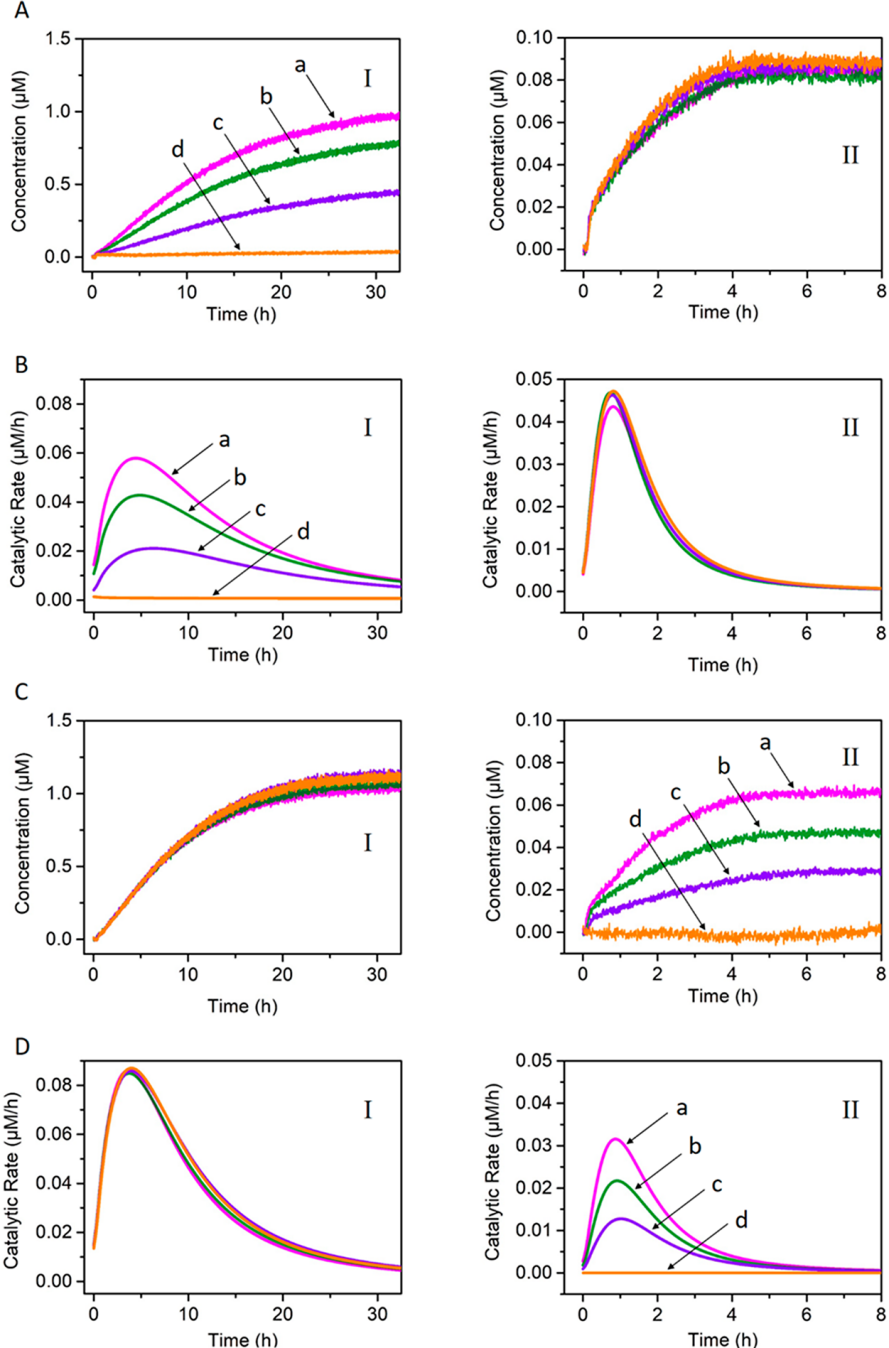
Concentration changes of the fragmented substrates and transient catalytic rates upon the inhibitor-guided gated operation of DNAzyme(**1**) and DNAzyme(**2**), according to Figure 6. (A) Time-dependent concentration changes of the fragmented fluorophore-generated substrates cleaved by the DNAzymes in state **2**: (panel I) DNAzyme(**1**), in the presence of variable concentrations of B_1_ = (a) 0.33, (b) 0.66, (c) 1, (d) 1.33 μM; (panel II) DNAzyme(**2**), in the presence of variable concentrations of B_1_ (shown in panel I). The DNAzyme(**2**) is unaffected by B_1_. (B) Transient catalytic rates corresponding to the DNAzymes in states **2**: (panel I) DNAzyme(**1**), in the presence of variable concentrations of B_1_ = (a) 0.33, (b) 0.66, (c) 1, (d) 1.33 μM; (panel II) DNAzyme(**2**) in the presence of the different concentrations of B_1_ outlined in panel I. (C) Time-dependent concentration changes of the fragmented fluorophore-generated substrates cleaved by the DNAzymes in state **3**: (panel I) DNAzyme(**1**), in the presence of variable concentrations of B_2_ outlined in panel II. The DNAzyme(**1**) is unaffected by B_2_. (panel II) DNAzyme(**2**) in the presence of the different concentrations of B_2_ = (a) 0.33, (b) 0.66, (c) 1, (d) 1.33 μM. (D) Transient catalytic rates corresponding to the DNAzymes in states **3**: (panel I) DNAzyme(**1**), in the presence of variable concentrations of B_2_ (shown in panel II); (panel II) DNAzyme(**2**) in the presence of the different concentrations of B_2_ = (a) 0.33, (b) 0.66, (c) 1, (d) 1.33 μM.

The inhibitor-guided gated catalytic activities of the mixture of DNAzymes are, also, introduced in Figure 6. Treatment of the constituents shown in state **1** with the inhibitor B_1_ leads to blockage of T_6_ (T_6_B_1_), resulting in the guided selective dimerization of the T_7_/T_8_ tetrahedra, and the selective activation of the Mg^2+^-ion-dependent DNAzyme(**2**), state **2**. Alternatively, subjecting state **1** to inhibitor B_2_, leads to the blockage of tetrahedron T_7_ (T_7_B_2_), resulting in the gated activation of the transient DNAzyme(**1**), state **3**. The gated and selective catalytic functions of DNAzyme(**1**) and DNAzyme(**2**) are demonstrated in Figure 8. In Figure 8A, the time-dependent concentration changes of the Cy5-labeled fragmented product generated by the DNAzyme(**1**), in the presence of variable concentrations of B_1_ are displayed in panel I, and the time-dependent concentration changes of the FAM-labeled fragmented product, generated by DNAzyme(**2**), at different concentrations of B_1_, are presented in panel II. The catalytic rates of DNAzyme(**1**) and DNAzyme(**2**), in the presence of variable concentrations of B_1_, are displayed in Figure 8B, panels I and II. As the concentration of B_1_ increases, the inhibition of formation of the Cy5-labeled fragment is higher, and at a B_1_ concentration of 1.33 μM, the formation of the Cy5-labeled fragmented product is fully blocked. At the same time, the time-dependent formation of the FAM-labeled fragmented product, generated by the DNAzyme(**2**), in the presence of variable concentrations of B_1_, is unaffected. Figure 8B depicts the gated, inhibitor-controlled, transient catalytic rates of DNAzyme(**1**), upon the addition of B_1_, panel I, and the lack of any inhibition effect on the transient catalytic rates of DNAzyme(**2**), panel II. Similarly, subjecting the tetrahedra mixture, state **1**, to the blocker strand B_2_ yields state **3**, where the tetrahedron T_7_ is blocked to form T_7_B_2_. Under these conditions, the dimerization of T_7_/T_8_ is blocked and the formation of DNAzyme(**2**) is inhibited. That is, B_2_ gates and guides the mixture of tetrahedra to selectively form the dimer T_5_/T_6_ and the accompanying DNAzyme(**1**) that cleaves substrate S_1_, leading to the transient formation of the Cy5-labeled fragmented product. Figure 8C, panels I and II, depicts the time-dependent concentration changes of the Cy5-modified fragmented product, generated by DNAzyme(**1**) and FAM-labeled fragmented product, generated by DNAzyme(**2**), in the presence of variable concentrations of the inhibitor B_2_, respectively. The formation of the Cy5-modified product generated by DNAzyme(**1**) is unaffected by the inhibitor B_2_, whereas the time-dependent formation of the FAM-labeled product is inhibited as the concentration of the inhibitor B_2_ is elevated, and, at a B_2_ concentration corresponding to 1.33 μM, the activity of DNAzyme(**2**) is fully blocked. In Figure 8D, panels I and II depict the catalytic rates of DNAzyme(**1**) and DNAzyme(**2**), derived from the time-dependent concentrations of Cy5- and FAM-modified products shown in Figure 8C. While no effect of B_2_ on the transient catalytic rates on the formation of Cy5-labeled product is observed, the transient catalytic rates corresponding to the formation of the FAM-labeled product are controlled by the concentration of B_2_, and as the concentration of B_2_ increases, the transient catalytic rates are decayed. The results are consistent with the selective inhibition of DNAzyme(**2**) and the gated activation of DNAzyme(**1**).

It should be noted that throughout the study, we applied DNA tetrahedra components as functional units that emulate small-sized proteins in operating transient dynamic dimerization and catalytic processes. Nonetheless, in principle, the transient dynamic transformations could be driven by duplex nucleic acid components that lack the tetrahedra substitutes. Accordingly, we wished to demonstrate that besides size-similarities between the tetrahedra and proteins, the tetrahedra subunits introduce protein-like functionalities into the structures that are nonexistent in analog “bare” duplexes. It was previously reported that catalytic units can be tethered to “inner” or “outer” positions of DNA tetrahedra, and the resulting catalyst tethers reveal different catalytic activities (albeit quite small yet reproducible differences).^31^ Accordingly, we designed DNA tetrahedra structures T_A_ and T_B_ that include hemin/G-quadruplex units in “inner” or “outer” positions and compared their activities to hemin/G-quadruplex tethered to a duplex DNA, Figure S17(A). The different hemin/G-quadruplex structures were examined toward two different catalytic transformations: (i) the hemin/G-quadruplex catalyzed oxidation of Amplex Red by H_2_O_2_ to form the fluorescent Resorufin, (ii) the hemin/G-quadruplex catalyzed oxidation of dopamine by H_2_O_2_ to form aminochrome. The results are summarized in Figure S17(B), panels I and II. The catalytic rates of the respective systems are summarized in Figure S17(C), panels I and II. The results demonstrate the improved catalytic functions of the catalytic units tethered to the tetrahedra, as compared to the “bare” hemin/G-quadruplex unit. Furthermore, the results show that the hemin/G-quadruplex units embedded in the tetrahedra reveal slightly enhanced catalytic activities as compared to the externally positioned catalyst. The inner functionalization of the tetrahedra with the catalyst mimics the embedding of catalytic sites in proteins. Thus, the results introduce the principles to enhance the complexity of transient protein-like assemblies by the functionalization of the tetrahedra subunits.

## Conclusions

The present study suggested DNA tetrahedra nanostructures as biomimetic analogs of small-sized proteins. The functional modification of the tetrahedra with complementary nucleic acids of catalytic nucleic acid sequences allowed the assembly of tetrahedra mixtures revealing transient formation and depletion. In the presence of pre-engineered auxiliary nucleic acid duplexes, inhibitors, the Nt.BbvCI nicking enzyme, and the DNA tetrahedra nanostructures, functional modules that guided transient dimerization and dynamic gated selective transient dimerization of DNA tetrahedra structures were demonstrated. In addition, by appropriate modification of the tetrahedra structures, the triggered gated emergence of selective dimer DNA tetrahedra nanostructures revealing transient catalytic properties was realized. We suggested the dynamic processes demonstrated the tetrahedra as model systems emulating the transient formation and depletion of protein–protein complexes and gated protein–protein structures in nature, and the transient emergence of guided catalytic functions as a result of protein–protein interactions in biological systems. We note, however, that, in principle, all dynamic processes described in our study could be performed by simple duplex nucleic acids that lack the tetrahedra conjugates. The significance of the tetrahedra nanostructures conjugated to the different systems rests, however, on the size resemblance between the tetrahedra units and small-sized proteins and the ability to embed protein-like functionalities and dictated catalytic functions in the tetrahedra structures in configurations that cannot be achieved by in analog duplex nucleic acids. In addition, the incorporation of the cell permeation elements^26,29,30^ into the dynamic assemblies allows the introduction of such systems into cellular environments. For example, as pointed out, the functionalization of the tetrahedra with the hemin/G-quadruplex DNAzyme units yields catalytic sites of enhanced activity as compared to the DNAzyme tethered to a duplex scaffold. Moreover, the tetrahedra scaffolds provide a means to embed the catalytic unit in “inner” or “outer” tetrahedra positions and tailor not only catalyst protein-like functionalities but also scaffolds exhibiting spatially dictated activities. Furthermore, the tetrahedra could be engineered to include tethers allowing the transient oligomerization of the tetrahedra and guided gated dimerization of the tetrahedra, similar to protein/protein transient binding and separation phenomenon. In addition, the modification of the tetrahedra with catalytic subunits allowed the transient emergence of catalytic functions in analogy to emerging catalytic properties of proteins guided by the transient oligomerization and separation of protein subunits. These analogies are, indeed, “Systems Chemistry” principles to mimic the structural complexity of biological systems and functions. In fact, the structural tetrahedra scaffolds allow the future engineering of biomimetic assemblies that cannot be realized by simple duplex nucleic acid structures. For example, we find that DNA tetrahedra units can be integrated with hydrogel microcapsules while duplex nucleic acids leak out from such carriers. This suggests that tetrahedra nanostructures could be encapsulated in cell-like containments, such as microdroplets^56^ or microcapsules.^57,58^ Thus, the minimal protein-like features of the tetrahedra units could allow the assembly of biomimetic protocells.

## Experimental Section

### Characterizations

The time-dependent fluorescence changes were followed at 33 °C on a Cary Eclipse Fluorometer (Varian Inc.). The excitations of Cy3, Cy5, FAM, and TAMRA were performed at 540, 540, 496, and 496 nm, respectively. The emissions of Cy3 Cy5, FAM, and TAMRA were recorded at 560, 660, 516, and 583 nm, respectively.

### Preparation of the DNA Tetrahedra

The DNA tetrahedron nanostructures (10 μM) were prepared by mixing equal amounts of four corresponding sequences, in 1× CutSmart buffer (50 mM Potassium Acetate, 20 mM Tris-acetate, 10 mM Magnesium Acetate, 100 μg mL^–1^ BSA, pH 7.9), and were heated to 90 °C for 5 min and then cooled down to 4 °C for 10 min.

### Preparation and Measurement of Dissipative Systems

Taking the first dissipative system (shown in Figure 1) as an example, 150 μL solutions were prepared with 15 μL of tetrahedron nanostructures (10 μM), 5 μL of duplex strands L_1_/I_1_ (30 μM), and 0.046 μM nicking enzyme (Nt.BbvCI, New England BioLabs Inc.). The prepared mixtures were subjected to different concentrations of fuel strands and time-dependent fluorescence changes were monitored spectroscopically at 33 °C. To study the effect of nicking enzyme, 150 μL solutions were prepared with 15 μL of tetrahedron nanostructures (10 μM), 5 μL of duplex strands L_1_/I_1_ (30 μM), and various concentrations of nicking enzyme (Nt.BbvCI, New England BioLabs Inc.). For the electrophoretic measurement, native PAGE (6%) was performed to characterize the time-dependent transient monomer/dimer at different time intervals and the band intensities of the gel image was analyzed by the ImageJ software, Figure S6.

### Measurements of the Gated Dissipative Systems

Equal amounts of four different tetrahedron structures were mixed (1 μM each, 150 μL) with the two duplexes L_1_I_1_, L_2_I_2_ (1 μM each), and 0.136 μM nicking enzyme (Nt.BbvCI, New England BioLabs Inc.) and with or without different concentrations of the corresponding inhibitor strands, B_1_ and B_2_. The prepared mixtures were added with the fuel strands L_1_′ and L_2_′ (4 μM each) and time-dependent fluorescence changes were monitored spectroscopically at 33 °C. The time-dependent fluorescence changes corresponding to the two tetrahedra dimers are followed by the evaluation of the FRET signals of fluorophore pairs of Cy3/Cy5 and FAM/TAMRA associated with the two tetrahedra dimers. Using the respective calibration curves of the two pairs of chromophores, Figures S3 and S7, the transient FRET signals were translated to transient concentration changes of the tetrahedra dimers T_1_/T_2_ and T_3_/T_4_. It should be noted, however, that the FRET signals of Cy3/Cy5 and FAM/TAMRA exhibit overlap features. To overcome this difficulty, each of the gating states shown in Figure 4 was characterized in two-separate analysis samples where one sample included the T_1_/T_2_ tetrahedra dimer labeled with Cy3/Cy5 and the other tetrahedra constituent, T_3_/T_4_, was nonlabeled. The second analysis sample included nonlabeled T_1_/T_2_ and the FAM/TAMRA-labeled T_3_/T_4_ constituent.

### Measurements of the Gated Dissipative Transient Catalytic Processes

Four different DNA tetrahedra extending with the corresponding DNAzyme subunits were prepared. Then equal amounts of these tetrahedron structures were mixed (0.5 μM each, 150 μL) with the two duplexes L_1_I_1_, L_2_I_2_ (0.5 μM each), 0.068 μM nicking enzyme (Nt.BbvCI, New England BioLabs Inc.), the substrates (3 μM S_1_, 2 μM S_2_) and with or without variable concentrations of the corresponding inhibitor strands, B_1_ and B_2_. The prepared mixtures were added with the fuel strands, L_1_′ and L_2_′ (2 μM each) and time-dependent fluorescence changes were monitored spectroscopically at 25 °C.

## Supporting Information Available

Materials, the DNA sequences, the calibration curves corresponding to the analyses of the system, the computational kinetic models formulated for the different transient systems, tables summarizing the rate-constants corresponding to the different models, the transient concentration changes of the tetrahedra T_3_/T_4_, and the fluorescence changes of the fragmented substrates of DNAzyme(**1**) and DNAzyme(**2**) (PDF)
