# Supplementary material for: Gated Transient Dissipative Dimerization of DNA Tetrahedra Nanostructures for Programmed DNAzymes Catalysis
Source: ACS Nano. 2022 Feb 20;16(3):3625–36. doi: 10.1021/acsnano.1c06117 (PMC8945371; doi:10.1021/acsnano.1c06117)
Supplement: Supplementary file 1 — nn1c06117_si_001.pdf [file nn1c06117_si_001.pdf]

Supporting Information

Gated Transient Dissipative Dimerization of DNA Tetrahedra Nanostructures for Programmed DNAzymes Catalysis

*Zhenzhen Li‡, Jianbang Wang‡, Zhixin Zhou‡, Michael P. O'Hagan & Itamar Willner**

The Institute of Chemistry, The Center for Nanoscience and Nanotechnology, The Hebrew University of Jerusalem, Jerusalem 91904, Israel.

‡These authors contributed equally to this study.

*E-mail: Itamar.willner@mail.huji.ac.il

Materials

Nicking enzyme (Nt.BbvCI, 10,000 units mL^{-1}, 2.3 μM) and CutSmart®Buffer were purchased from New England BioLabs Inc.. Oligonucleotides were purchased from Integrated DNA Technologies. The Cy3-, Cy5-, FAM- and TAMRA-labeled oligonucleotides were purified by high-performance liquid chromatography. All the sequences of the oligonucleotides were listed below.

A13: 5'-ACACTACGTCAGAACAGCTTGCATCACTGGTCACCAGAGTA-3'

B13: 5'-ACGAGCGAGTTGATGTGATGCAAGCTGAATGCGAGGGTCCT-3'

C13: 5'-TCAACTCGCTCGTAACTACACTGTGCAATACTCTGGTGACC-3'

D13: 5'-TCTGACGTAGTGTATGCACAGTGTAGTAAGGACCCTCGCAT-3'

B7: 5'-TTAGGCGAGTGTGGCAGAGGTGT-3'

C7: 5'-CGCCTAAACAAGTGGAGACTGTG-3'

D7: 5'-AACGCTCACCACTTGAACACCTC-3'

A13-CY3: 5'-CY3-CCATCACAAAAACAAAACACTACGTCAGAACAGCTTGCATCACTGG TCACCAGAGTA-3'

A13-CY5: 5'-ACACTACGTCAGAACAGCTTGCATCACTGGTCACCAGAGTAAAAACACA GAAGAAC-CY5-3'

C13-FAM: 5'-TCAACTCGCTCGTAACTACACTGTGCAATACTCTGGTGACCAAAAAAAGA CAAAGGA-FAM-3'

A7-TAMARA: 5'-TAMARA-GACACAACAAAAAAAAGAGCGTTAGCCACACACACAGTC-3'

I_1: 5'-GGTTTGTGATGGACGTTCTTCTGTC-3'

L_1: 5'-GACAGAAGAACGCTGAGGCCATCACAAACC-3'

L_1': 5'-GATGGCCTCAGCGTT-3'

I_2: 5'-CGTTGTTGTGTCACTCCTTTGTCTG-3'

L_2: 5'-CAGACAAAGGAGCTGAGGGACACAACAACG-3'

L_2': 5'-GTGTCCCTCAGCTCC-3'

B_1: 5'-TTTGTTTTTGTGATG-3'

B_2: 5'-CCTTTGTCTTTTTTT-3'

A13-E1: 5'-GTCCTCAGCGATGTTACCATCACAAAAACAAAACACTACGTCAGAACAGCT

TGCATCACTGGTCACCAGAGTA-3’

A13-E2: 5’-ACACTACGTCAGAACAGCTTGCATCACTGGTCACCAGAGTAAAAACACAG AAGAACTAACCACCCATGTTTCAGT-3’

C13-E3: 5’-TCAACTCGCTCGTAACTACACTGTGCAATACTCTGGTGACCAAAAAA AGAC AAAGGATAACCACCCATGTTCCTGA-3’

A7-E4: 5’-CTGTTCAGCGATGTTAGACACAACAAAAAAAAGAGCGTTAGCCACACACAC AGTC-3’

S_1: 5’-Cy5-ACTGAA TrAG GAGGAC-BHQ2-3’

S_2: 5’-FAM-TCAGGA TrAG GAACAG-IBRQ-3’

T_1: 5’-CTTGCTACACGATTCAGACTTAGGAATGTTCGACATGCGAGGGTCCAATACCGAC GATTACAG-3’

T_2: 5’-GGTGATAAAACGTGTAGCAAGCTGTAATCGACGGGAAGAGCATGCCCATCCACT ACTATGGCG-3’

T_3: 5’-CCTCGCATGACTCAACTGCCTGGTGATACGAGGATGGGCATGCTCTTCCCGACGG TATTGGAC-3’

T_{4}-inner: 5’-*GGGTAGGGCGGGTTGGG* CCAGGCAGTTGAGACGAACATTCCTAAGTCTGA AATTTATCACCCGCCATAGTAGACGTATCA-3’

T_{4}-outer: 5’-*GGGTAGGGCGGGTTGGG* CAGTTGAGACGAACATTCCTAAGTCTGAAATT TATCACCCGCCATAGTAGACGTATCACCAGG-3’

G: 5’-*GGGTAGGGCGGGTTGGG* CAGCATAGAATCCAGG-3’

C_G: 5’-CCTGGATTCTATGCTG-3’

The principles to design such transient system should include the following rules:

-The stability of the different duplexes involved in the reaction module are determined by the number and nature of base pairs. The energetic stabilization of the duplex is estimated using NUPACK software.

-The energetic stabilization of the rest reaction module is controlled by the energy level of the duplex L_1/I_1.

-The triggered transition of the rest module to the transient T_1/T_2 tetrahedra structure requires the intermediate products L_1/L_1' and I_1/T_1T_2 dimer that should be energetically more stable than L_1/I_1.

-The cleavage of the duplex L_1/L_1' separates the "waste" products of the fragmented pieces of L_1'. The released L_1 displaces I_1 from the dimer I_1/T_1T_2 guided by the enhanced energetic stability of L_1/I_1 as compared to I_1/T_1T_2.

The following scheme outlines the relative energy levels of the constituents associated with the transient operation of the network.

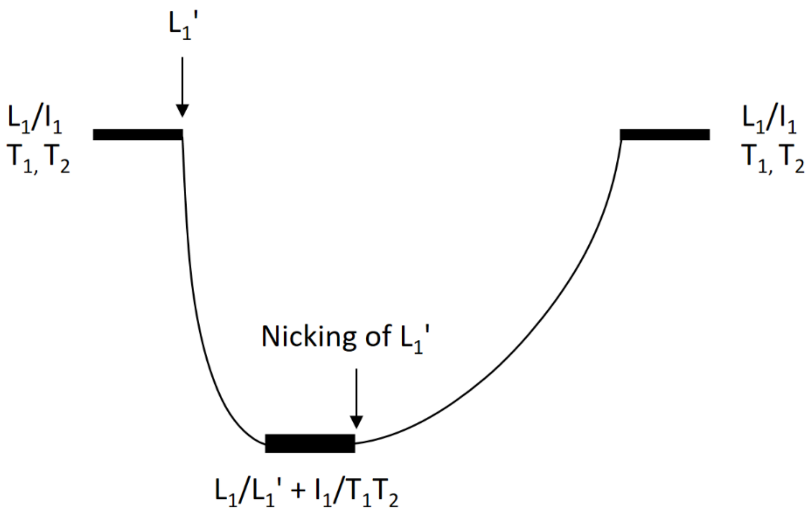

Figure S1. Scheme of the relative energy levels of the constituents associated with the transient operation of the network.

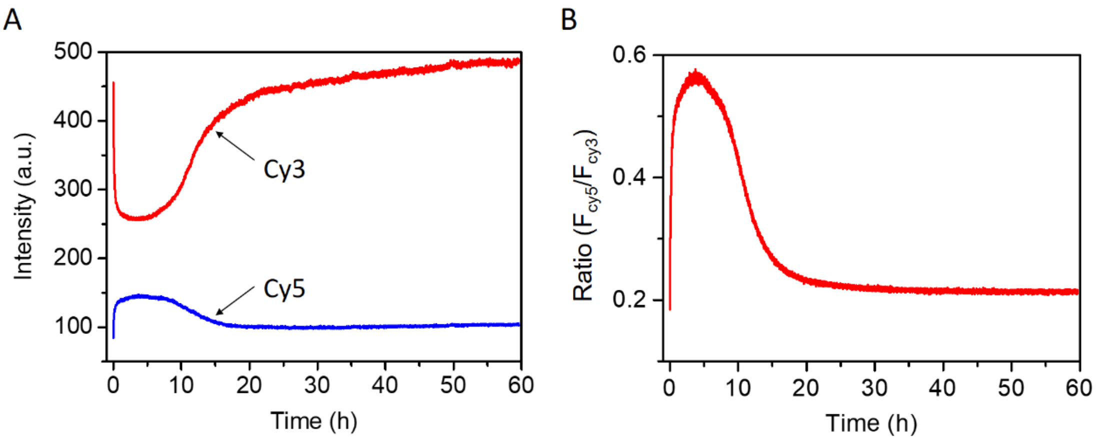

Figure S2. (A) Time-dependent fluorescence changes of Cy3 and Cy5 that follow the dynamic transient formation and depletion of the dimer structure T_1/T_2. (B) The ratio F_{Cy5}/F_{Cy3} follows the formation and depletion of the dimer structure T_1/T_2.

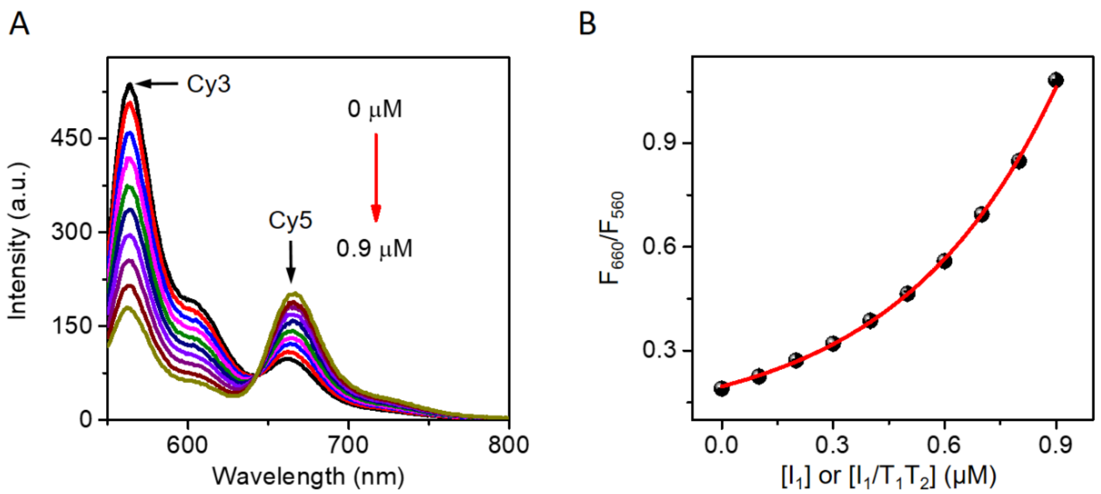

Figure S3. (A) The fluorescence spectra of T_1 and T_2 upon the addition of different concentrations of I_1. (B) The derived calibration curve corresponding to the ratio of the intensities of Cy5 and Cy3, F_{660}/F_{560}, at different concentrations of I_1, yielding the supramolecular complex I_1/T_1T_2 (means ± standard deviation, n = 3).

It should be noted that the format of plotting the calibration curve yields a non-linear increase. This can be quantitatively rationalized as follows:

For this low concentration (1 μM) system, the fluorescence intensities of Cy3 (F_1) and Cy5 (F_2) are given by equation (i) and equation (ii):

(i) $F_1 = \alpha_1 * C_{cy3}$

(ii) $F_2 = \alpha_2 * C_{cy5/cy3}$

where Cy5/Cy3 corresponds to the concentration of Cy5 in the FRET luminescent complex composed of I_1/T_1T_2.

Since the start concentration of Cy3 is 1μM, the transient concentration of Cy3 is given by equation (iii)

(iii) $C_{cy3} = 1 - C_{cy5/cy3}$

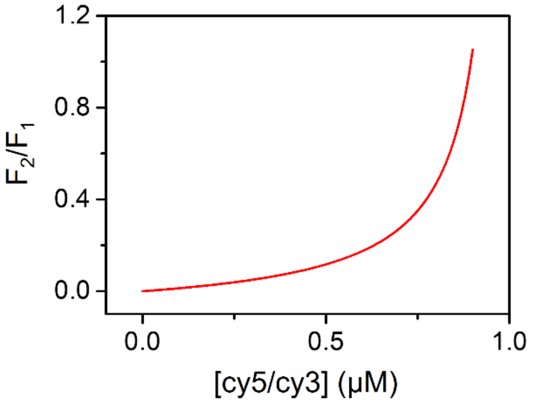

Figure S3(C)

Thus, the ratio of F_2/F_1 is given by equation (iv) that can be rewritten as follows:

$$\text{(iv)}\quad \frac{F_2}{F_1} = \frac{\alpha_2}{\alpha_1} * \frac{C_{cy5/cy3}}{C_{cy3}} = \frac{\alpha_2}{\alpha_1} * \frac{C_{cy5/cy3}}{1\text{-}C_{cy5/cy3}}$$

That is, the ratio of F_2/F_1 follows a classical parabolic function $f = k * \frac{x}{1-x}$ as depicted in Figure S3(C) (k set as 0.117).

Kinetic equations of the dissipative Cy5/Cy3 FRET system shown in Figure 1:

$$L_1 + I_1 \underset{k_{-1}}{\overset{k_1}{\rightleftharpoons}} L_1I_1 \qquad (1)$$

$$L_1I_1 + L_1' \underset{k_{-2}}{\overset{k_2}{\rightleftharpoons}} L_1L_1' + I_1 \qquad (2)$$

$$T_1 + T_2 + I_1 \underset{k_{-3}}{\overset{k_3}{\rightleftharpoons}} T_1T_2I_1 \qquad (3)$$

$$L_1L_1' + E \underset{k_{-4}}{\overset{k_4}{\rightleftharpoons}} L_1L_1'E \qquad (4)$$

$$L_1L_1'E \xrightarrow{k_5} E + L_1L_{1\text{-}1}'L_{1\text{-}2}' \qquad (5)$$

$$L_1L_{1\text{-}1}'L_{1\text{-}2}' \underset{k_{-6}}{\overset{k_6}{\rightleftharpoons}} L_1 + L_{1\text{-}1}' + L_{1\text{-}2}' \qquad (6)$$

Derivatives:

$$\frac{dL_1}{dt} = -k_1[L_1][I_1] + k_{-1}[L_1I_1] + k_6[L_1L_{1\text{-}1}'L_{1\text{-}2}'] - k_{-6}[L_1][L_{1\text{-}1}'][L_{1\text{-}2}']$$

$$\frac{dI_1}{dt} = -k_1[L_1][I_1] + k_{-1}[L_1I_1] + k_2[L_1I_1][L_1'] - k_{-2}[L_1L_1'][I_1] - k_3[T_1][T_2][I_1] + k_{-3}[T_1T_2I_1]$$

$$\frac{dL_1I_1}{dt} = k_1[L_1][I_1] - k_{-1}[L_1I_1] - k_2[L_1I_1][L_1'] + k_{-2}[L_1L_1'][I_1]$$

$$\frac{dL_1'}{dt} = -k_2[L_1I_1][L_1'] + k_{-2}[L_1L_1'][I_1]$$

$$\frac{dL_1L_1'}{dt} = k_2[L_1I_1][L_1'] - k_{-2}[L_1L_1'][I_1] - k_4[L_1L_1'][E] + k_{-4}[L_1L_1'E]$$

$$\frac{dT_1}{dt} = -k_3[T_1][T_2][I_1] + k_{-3}[T_1T_2I_1]$$

$$\frac{dT_2}{dt} = -k_3[T_1][T_2][I_1] + k_{-3}[T_1T_2I_1]$$

$$\frac{dT_1T_2I_1}{dt} = k_3[T_1][T_2][I_1] - k_{-3}[T_1T_2I_1]$$

$$\frac{dE}{dt} = -k_4[L_1L_1'][E] + k_{-4}[L_1L_1'E] + k_5[L_1L_1'E]$$

$$\frac{dL_1L_1'E}{dt} = k_4[L_1L_1'][E] - k_{-4}[L_1L_1'E] - k_5[L_1L_1'E]$$

$$\frac{dL_1L_{1-1}'L_{1-2}'}{dt} = k_5[L_1L_1'E] - k_6[L_1L_{1\text{-}1}'L_{1\text{-}2}'] + k_{-6}[L_1][L_{1\text{-}1}'][L_{1\text{-}2}']$$

$$\frac{dL_{1-1}'}{dt} = k_6[L_1L_{1\text{-}1}'L_{1\text{-}2}'] - k_{-6}[L_1][L_{1\text{-}1}'][L_{1\text{-}2}']$$

$$\frac{dL_{1-2}'}{dt} = k_6[L_1L_{1\text{-}1}'L_{1\text{-}2}'] - k_{-6}[L_1][L_{1\text{-}1}'][L_{1\text{-}2}']$$

Figure S4. Computational simulation of the dissipative Cy5/Cy3 FRET system shown in Figure 1. The kinetic scheme of the reactions associated with the time-dependent concentration changes during the dissipative transitions of FRET is summarized in above equations. Knowing the time-dependent concentration changes of the tetrahedron dimers associated with T_1/T_2, during the dissipative transitions of FRET, we computationally simulated the time-dependent concentration changes by using Matlab R2020a.

It is important to note that the computationally simulated rate constants have a meaning only if one or more of the rate-constants can be evaluated independently experiment and computationally, and the result should be compared to the analogy rate-constant being part of the overall kinetic model. According the values of k_3 and k_{-3} in the presence of two concentrations of I_1 were evaluated as follows, Figure S5.

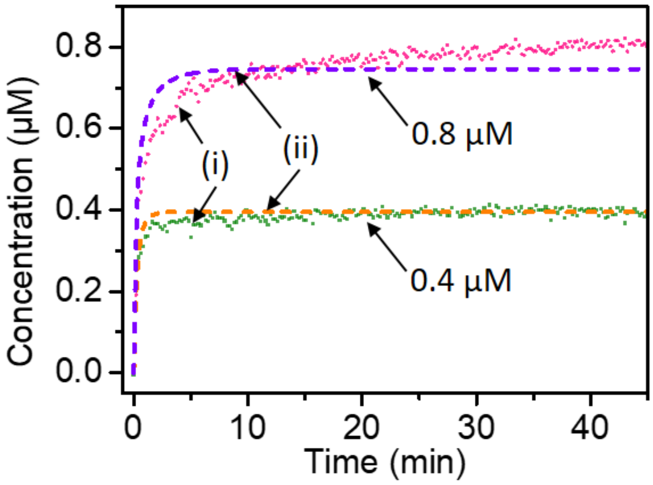

Figure S5. Time-dependent concentration changes of T_1/T_2 complex upon subjecting tetrahedra T_1 and T_2 to different concentrations of I_1: 0.4 μM and 0.8 μM. Dashed curves correspond to the computationally simulated kinetic profiles. Dotted curves represent the experimental data.

Experimental (i)-dotted lines and computationally (ii)-dashed lines

Evaluation of the rate constants k_3 and k_{-3} of the kinetic scheme in Figure S4.

To evaluate the rate constants k_3 and k_{-3} appearing in the kinetic scheme, equation (3) in Figure S4, the mixture of T_1 and T_2 (1 μM each) was subjected to two different concentrations of I_1 (0.4 μM and 0.8 μM), and the time-dependent concentration changes were evaluated by following the time-dependent fluorescence changes of the tetrahedron dimers associated with T_1/T_2, and translating them into concentrations by applying an appropriate calibration curve, Figure S3. From the kinetic profiles and using the Matlab R2020a program, the respective $k_3 = 5\ \mu M^{-2}\ min^{-1}$ and $k_{-3} = 0.0236\ min^{-1}$ were derived. These values fit well with the computationally simulated rate constant following the overall kinetic model and Table S1.

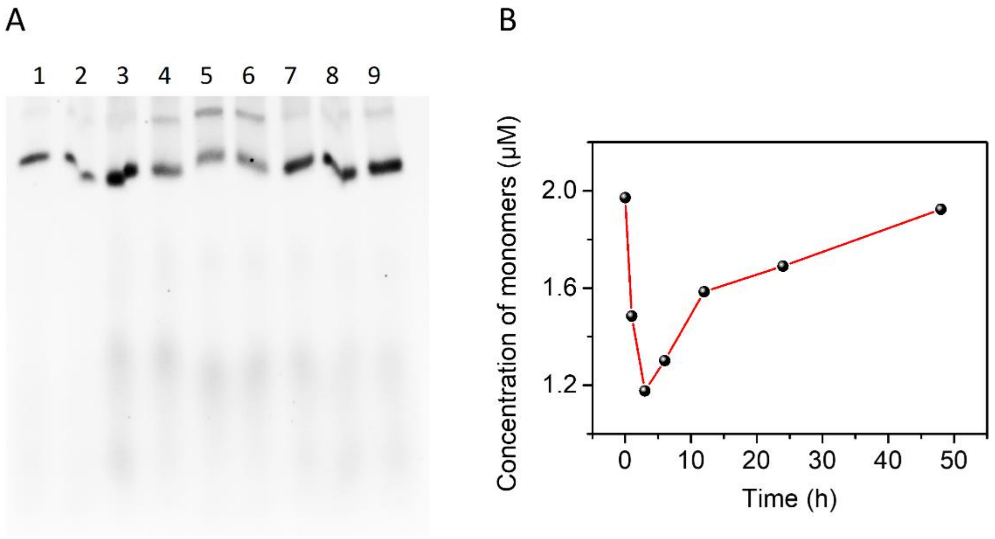

Figure S6. Time-dependent gel electrophoretic characterization of the transient T_1, T_2 dimerization to T_1-T_2 according to Figure 1 upon the L_1'-triggered transient dimerization of the tetrahedra monomers. (A) Gel electrophoretic separated bands corresponding to the tetrahedra monomer (lower band) and T_1/T_2 tetrahedra dimer (upper bands). Bottom bands: lane1 T_1, lane 2 T_2, lane 3 to lane 9 composition of the tetrahedra T_1 and T_2 triggered by L_1' at different times of the transient process. Lane 3 at t = 0; lane 4 at t = 1 h; lane 5 at t = 3 h; lane 6 at t = 6 h; lane 7 at t = 12 h; lane 8 at t = 24 h; lane 9 at t = 48 h. The samples withdrawn from the reaction mixture were treated with trypsin to digest the nicking enzyme and retain the respective time-dependent composition of the transient dimerization process prior to the electrophoretic separation. (B) Concentrations of the monomer mixture T_1, T_2 along the transient dimerization process. Due to the relatively low concentrations of the dimer T_1/T_2, the dissipative, transient, evaluation of the process was followed by quantitative analysis of the stained mixture of the T_1/T_2 monomer constituents along the transient process.

The quantitative evaluation of the transient electrophoretic separated monomers T_1/T_2 mixtures was performed by measuring the intensities of the stained bands using the ImageJ software and comparing the results the stained bands of T_1/T_2 at different known concentrations.

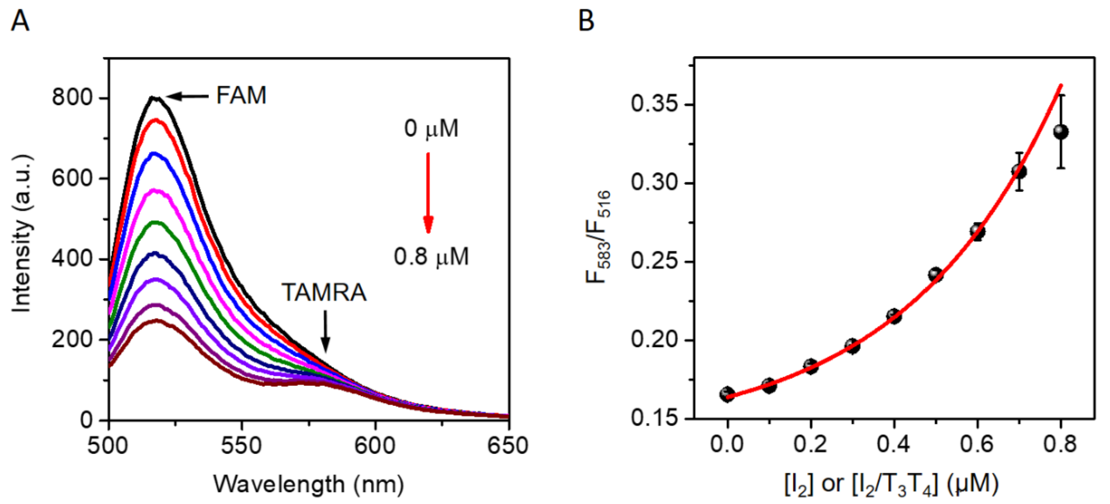

Figure S7. (A) The fluorescence spectra of T_3 and T_4 upon the addition of different concentrations of I_2. (B) The derived calibration curve corresponding to the ratio of the intensities of TAMRA and FAM, F_{583}/F_{516}, at different concentrations of I_2, yielding the supramolecular complex I_2/T_3T_4 (means ± standard deviation, n = 3).

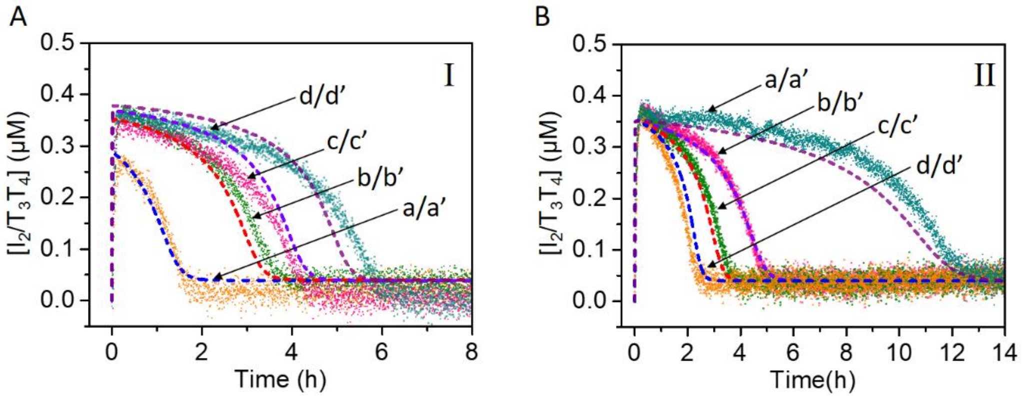

Figure S8. (A) Transient concentration changes of the tetrahedra T_3/T_4 upon the fuel L_2’ triggered formation and depletion of the T_3/T_4 dimer in the presnce of variable concentrations of the fuel strand L_2’: (a) = 2 μM, (b) = 4 μM, (c) = 6 μM, (d) = 8 μM. For all systems: L_2/I_2= 1 μM; T_3= 1 μM; T_4 = 1 μM and Nt.BbvCI 0.046 μM. (Dotted lines correspond to experimental results. Dashed curves a’, b’, c’, d’ correspond to the computational simulated transient, using the kinetic model presented in Figure S9). Curves c/c’ correspond to the experimental transient (dotted line) followed by the computational simulation (dashed line), using the kinetic model, leading to the derived rate constants tabulated in Table S2. Curves a/a’, b/b’ and d/d’ were first computationally simulated and subsequently experimentally validated. (B) Transient concentration changes of the tetrahedra T_3/T_4 upon the fuel L_2’ triggered formation and depletion of the T_3/T_4 dimer in the presence of variable concentrations of Nt.BbvCI: (a) = 0.0153 μM, (b) = 0.0306 μM, (c) = 0.046 μM, (d) = 0.0612 μM. For all systems: L_2/I_2 = 1 μM; T_3= 1 μM; T_4 = 1 μM and fuel strand L_2’ = 4 μM. (Dotted lines correspond to experimental results. Dashed curves a’, b’, c’ and d’ correspond to the computational simulated transients, using the kinetic model presented in Figure S9). Curves a/a’, b/b’, c/c’ and d/d’ were first computationally simulated and subsequently experimentally validated.

Kinetic equations of the dissipative TAMRA/FAM FRET system shown in Figure 3:

$$L_2 + I_2 \underset{k_{-7}}{\overset{k_7}{\rightleftharpoons}} L_2I_2 \quad (7)$$

$$L_2I_2 + L_2' \underset{k_{-8}}{\overset{k_8}{\rightleftharpoons}} L_2L_2' + I_2 \quad (8)$$

$$T_3 + T_4 + I_2 \underset{k_{-9}}{\overset{k_9}{\rightleftharpoons}} T_3T_4I_2 \quad (9)$$

$$L_2L_2' + E \underset{k_{-4}}{\overset{k_4}{\rightleftharpoons}} L_2L_2'E \quad (10)$$

$$L_2L_2'E \xrightarrow{k_5} E + L_2L_{2\text{-}1}'L_{2\text{-}2}' \quad (11)$$

$$L_2L_{2\text{-}1}'L_{2\text{-}2}' \underset{k_{-10}}{\overset{k_{10}}{\rightleftharpoons}} L_2 + L_{2\text{-}1}' + L_{2\text{-}2}' \quad (12)$$

Derivatives:

$$\frac{dL_2}{dt} = -k_7[L_2][I_2] + k_{-7}[L_2I_2] + k_{10}[L_2L_{2\text{-}1}'L_{2\text{-}2}'] - k_{-10}[L_2][L_{2\text{-}1}'][L_{2\text{-}2}']$$

$$\frac{dI_2}{dt} = -k_7[L_2][I_2] + k_{-7}[L_2I_2] + k_8[L_2I_2][L_2'] - k_{-8}[L_2L_2'][I_2] - k_9[T_3][T_4][I_2] + k_{-9}[T_3T_4I_2]$$

$$\frac{dL_2I_2}{dt} = k_7[L_2][I_2] - k_{-7}[L_2I_2] - k_8[L_2I_2][L_2'] + k_{-8}[L_2L_2'][I_2]$$

$$\frac{dL_2'}{dt} = -k_8[L_2I_2][L_2'] + k_{-8}[L_2L_2'][I_2]$$

$$\frac{dL_2L_2'}{dt} = k_8[L_2I_2][L_2'] - k_{-8}[L_2L_2'][I_2] - k_4[L_2L_2'][E] + k_{-4}[L_2L_2'E]$$

$$\frac{dT_3}{dt} = -k_9[T_3][T_4][I_2] + k_{-9}[T_3T_4I_2]$$

$$\frac{dT_4}{dt} = -k_9[T_3][T_4][I_2] + k_{-9}[T_3T_4I_2]$$

$$\frac{dT_3T_4I_2}{dt} = k_9[T_3][T_4][I_2] - k_{-9}[T_3T_4I_2]$$

$$\frac{dE}{dt} = -k_4[L_2L_2'][E] + k_{-4}[L_2L_2'E] + k_5[L_2L_2'E]$$

$$\frac{dL_2L_2'E}{dt} = k_4[L_2L_2'][E] - k_{-4}[L_2L_2'E] - k_5[L_2L_2'E]$$

$$\frac{dL_2L_{2-1}'L_{2-2}'}{dt} = k_5[L_2L_2'E] - k_{10}[L_2L_{2\text{-}1}'L_{2\text{-}2}'] + k_{-10}[L_2][L_{2\text{-}1}'][L_{2\text{-}2}']$$

$$\frac{dL_{2-1}'}{dt} = k_{10}[L_2L_{2\text{-}1}'L_{2\text{-}2}'] - k_{-10}[L_2][L_{2\text{-}1}'][L_{2\text{-}2}']$$

$$\frac{dL_{2-2}'}{dt} = k_{10}[L_2L_{2\text{-}1}'L_{2\text{-}2}'] - k_{-10}[L_2][L_{2\text{-}1}'][L_{2\text{-}2}']$$

Figure S9. Computational simulation of the dissipative TAMRA/FAM FRET system shown in Figure 3. The kinetic scheme of the reactions associated with the time-dependent concentration changes during the dissipative transitions of FRET is summarized in above equations. Knowing the time-dependent concentration changes of the tetrahedron dimers associated with T_3/T_4, during the dissipative transitions of FRET, we computationally simulated the time-dependent concentration changes by using Matlab R2020a.

It is important to note that the computationally simulated rate constants have a meaning only if one or more of the rate-constants can be evaluated independently experiment and computationally, and the result should be compared to the analogy rate-constant being part of the overall kinetic model. According the values of k_9 and k_{-9} in the presence of two concentrations of I_2 were evaluated as follows, Figure S10.

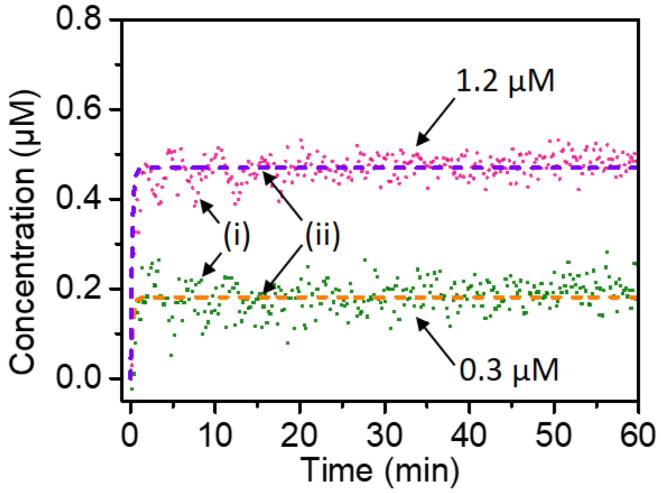

Figure S10. Time-dependent concentration changes of T_3/T_4 complex upon subjecting tetrahedra, T_3 and T_4, to different concentrations of I_2: 0.3 µM and 1.2 µM. Dashed curves correspond to the computationally simulated kinetic profiles. Dotted curves represent the experimental data.

Experimental (i)-dotted lines and computationally (ii)-dashed lines

Evaluation of the rate constants k_9 and k_{-9} of the kinetic scheme in Figure S9.

To evaluate the rate constants k_9 and k_{-9} appearing in the kinetic scheme, equation (9) in Figure S9, the mixture of T_3 and T_4 (1 µM each) was subjected to two different concentrations of I_2 (0.3 µM and 1.2 µM), and the time-dependent concentration changes were evaluated by following the time-dependent fluorescence changes of the tetrahedron dimers associated with T_3/T_4, and translating them into contents by applying an appropriate calibration curve, Figure S7. From the kinetic profiles and using the Matlab R2020a program, the respective $k_9 = 2.985$ µM^{-2} min^{-1} and $k_{-9} = 1.31$ min^{-1} were derived.

Kinetic equations of the gated dissipative system (Figure 4, State A):

$$L_1 + I_1 \underset{k_{-1}}{\overset{k_1}{\rightleftharpoons}} L_1I_1 \quad (1)$$

$$L_1I_1 + L_1' \underset{k_{-2}}{\overset{k_2}{\rightleftharpoons}} L_1L_1' + I_1 \quad (2)$$

$$T_1 + T_2 + I_1 \underset{k_{-3}}{\overset{k_3}{\rightleftharpoons}} T_1T_2I_1 \quad (3)$$

$$L_1L_1' + E \underset{k_{-4}}{\overset{k_4}{\rightleftharpoons}} L_1L_1'E \quad (4)$$

$$L_1L_1'E \xrightarrow{k_5} E + L_1L_{1\text{-}1}'L_{1\text{-}2}' \quad (5)$$

$$L_1L_{1\text{-}1}'L_{1\text{-}2}' \underset{k_{-6}}{\overset{k_6}{\rightleftharpoons}} L_1 + L_{1\text{-}1}' + L_{1\text{-}2}' \quad (6)$$

$$L_2 + I_2 \underset{k_{-7}}{\overset{k_7}{\rightleftharpoons}} L_2I_2 \quad (7)$$

$$L_2I_2 + L_2' \underset{k_{-8}}{\overset{k_8}{\rightleftharpoons}} L_2L_2' + I_2 \quad (8)$$

$$T_3 + T_4 + I_2 \underset{k_{-9}}{\overset{k_9}{\rightleftharpoons}} T_3T_4I_2 \quad (9)$$

$$L_2L_2' + E \underset{k_{-4}}{\overset{k_4}{\rightleftharpoons}} L_2L_2'E \quad (10)$$

$$L_2L_2'E \xrightarrow{k_5} E + L_2L_{2\text{-}1}'L_{2\text{-}2}' \quad (11)$$

$$L_2L_{2\text{-}1}'L_{2\text{-}2}' \underset{k_{-10}}{\overset{k_{10}}{\rightleftharpoons}} L_2 + L_{2\text{-}1}' + L_{2\text{-}2}' \quad (12)$$

Derivatives:

$$\frac{dL_1}{dt} = -k_1[L_1][I_1] + k_{-1}[L_1I_1] + k_6[L_1L_{1\text{-}1}'L_{1\text{-}2}'] - k_{-6}[L_1][L_{1\text{-}1}'][L_{1\text{-}2}']$$

$$\frac{dI_1}{dt} = -k_1[L_1][I_1] + k_{-1}[L_1I_1] + k_2[L_1I_1][L_1'] - k_{-2}[L_1L_1'][I_1] - k_3[T_1][T_2][I_1] + k_{-3}[T_1T_2I_1]$$

$$\frac{dL_1I_1}{dt} = k_1[L_1][I_1] - k_{-1}[L_1I_1] - k_2[L_1I_1][L_1'] + k_{-2}[L_1L_1'][I_1]$$

$$\frac{dL_1'}{dt} = -k_2[L_1I_1][L_1'] + k_{-2}[L_1L_1'][I_1]$$

$$\frac{dL_1L_1'}{dt} = k_2[L_1I_1][L_1'] - k_{-2}[L_1L_1'][I_1] - k_4[L_1L_1'][E] + k_{-4}[L_1L_1'E]$$

$$\frac{dT_1}{dt} = -k_3[T_1][T_2][I_1] + k_{-3}[T_1T_2I_1]$$

$$\frac{dT_2}{dt} = -k_3[T_1][T_2][I_1] + k_{-3}[T_1T_2I_1]$$

$$\frac{dT_1T_2I_1}{dt} = k_3[T_1][T_2][I_1] - k_{-3}[T_1T_2I_1]$$

$$\frac{dE}{dt} = -k_4[L_1L_1'][E] + k_{-4}[L_1L_1'E] + k_5[L_1L_1'E] - k_4[L_2L_2'][E] + k_{-4}[L_2L_2'E] + k_5[L_2L_2'E]$$

$$\frac{dL_1L_1'E}{dt} = k_4[L_1L_1'][E] - k_{-4}[L_1L_1'E] - k_5[L_1L_1'E]$$

$$\frac{dL_1L_{1-1}'L_{1-2}'}{dt} = k_5[L_1L_1'E] - k_6[L_1L_{1\text{-}1}'L_{1\text{-}2}'] + k_{-6}[L_1][L_{1\text{-}1}'][L_{1\text{-}2}']$$

$$\frac{dL_{1-1}'}{dt} = k_6[L_1L_{1\text{-}1}'L_{1\text{-}2}'] - k_{-6}[L_1][L_{1\text{-}1}'][L_{1\text{-}2}']$$

$$\frac{dL_{1-2}'}{dt} = k_6[L_1L_{1\text{-}1}'L_{1\text{-}2}'] - k_{-6}[L_1][L_{1\text{-}1}'][L_{1\text{-}2}']$$

$$\frac{dL_2}{dt} = -k_7[L_2][I_2] + k_{-7}[L_2I_2] + k_{10}[L_2L_{2\text{-}1}'L_{2\text{-}2}'] - k_{-10}[L_2][L_{2\text{-}1}'][L_{2\text{-}2}']$$

$$\frac{dI_2}{dt} = -k_7[L_2][I_2] + k_{-7}[L_2I_2] + k_8[L_2I_2][L_2'] - k_{-8}[L_2L_2'][I_2] - k_9[T_3][T_4][I_2] + k_{-9}[T_3T_4I_2]$$

$$\frac{dL_2I_2}{dt} = k_7[L_2][I_2] - k_{-7}[L_2I_2] - k_8[L_2I_2][L_2'] + k_{-8}[L_2L_2'][I_2]$$

$$\frac{dL_2'}{dt} = -k_8[L_2I_2][L_2'] + k_{-8}[L_2L_2'][I_2]$$

$$\frac{dL_2L_2'}{dt} = k_8[L_2I_2][L_2'] - k_{-8}[L_2L_2'][I_2] - k_4[L_2L_2'][E] + k_{-4}[L_2L_2'E]$$

$$\frac{dT_3}{dt} = -k_9[T_3][T_4][I_2] + k_{-9}[T_3T_4I_2]$$

$$\frac{dT_4}{dt} = -k_9[T_3][T_4][I_2] + k_{-9}[T_3T_4I_2]$$

$$\frac{dT_3T_4I_2}{dt} = k_9[T_3][T_4][I_2] - k_{-9}[T_3T_4I_2]$$

$$\frac{dL_2L_2'E}{dt} = k_4[L_2L_2'][E] - k_{-4}[L_2L_2'E] - k_5[L_2L_2'E]$$

$$\frac{dL_2L_{2-1}'L_{2-2}'}{dt} = k_5[L_2L_2'E] - k_{10}[L_2L_{2-1}'L_{2-2}'] + k_{-10}[L_2][L_{2-1}'][L_{2-2}']$$

$$\frac{dL_{2-1}'}{dt} = k_{10}[L_2L_{2-1}'L_{2-2}'] - k_{-10}[L_2][L_{2-1}'][L_{2-2}']$$

$$\frac{dL_{2-2}'}{dt} = k_{10}[L_2L_{2-1}'L_{2-2}'] - k_{-10}[L_2][L_{2-1}'][L_{2-2}']$$

Figure S11. Computational simulation of the gated dissipative system (Figure 4, State **A**). The kinetic scheme of the reactions associated with the time-dependent concentration changes during the dissipative transitions is summarized in above equations. Knowing the time-dependent concentration changes of the tetrahedron dimers T_1/T_2 and T_3/T_4 during the dissipative transitions, we computationally simulated the time-dependent concentration changes by using Matlab R2020a.

Kinetic equations of B_1 gated dissipative system (Figure 4, State B):

$$L_1 + I_1 \underset{k_{-1}}{\overset{k_1}{\rightleftharpoons}} L_1I_1 \quad (1)$$

$$L_1I_1 + L_1' \underset{k_{-2}}{\overset{k_2}{\rightleftharpoons}} L_1L_1' + I_1 \quad (2)$$

$$T_1 + T_2 + I_1 \underset{k_{-3}}{\overset{k_3}{\rightleftharpoons}} T_1T_2I_1 \quad (3)$$

$$L_1L_1' + E \underset{k_{-4}}{\overset{k_4}{\rightleftharpoons}} L_1L_1'E \quad (4)$$

$$L_1L_1'E \xrightarrow{k_5} E + L_1L_{1\text{-}1}'L_{1\text{-}2}' \quad (5)$$

$$L_1L_{1\text{-}1}'L_{1\text{-}2}' \underset{k_{-6}}{\overset{k_6}{\rightleftharpoons}} L_1 + L_{1\text{-}1}' + L_{1\text{-}2}' \quad (6)$$

$$T_1 + B_1 \underset{k_{-11}}{\overset{k_{11}}{\rightleftharpoons}} T_1B_1 \quad (13)$$

$$L_2 + I_2 \underset{k_{-7}}{\overset{k_7}{\rightleftharpoons}} L_2I_2 \quad (7)$$

$$L_2I_2 + L_2' \underset{k_{-8}}{\overset{k_8}{\rightleftharpoons}} L_2L_2' + I_2 \quad (8)$$

$$T_3 + T_4 + I_2 \underset{k_{-9}}{\overset{k_9}{\rightleftharpoons}} T_3T_4I_2 \quad (9)$$

$$L_2L_2' + E \underset{k_{-4}}{\overset{k_4}{\rightleftharpoons}} L_2L_2'E \quad (10)$$

$$L_2L_2'E \xrightarrow{k_5} E + L_2L_{2\text{-}1}'L_{2\text{-}2}' \quad (11)$$

$$L_2L_{2\text{-}1}'L_{2\text{-}2}' \underset{k_{-10}}{\overset{k_{10}}{\rightleftharpoons}} L_2 + L_{2\text{-}1}' + L_{2\text{-}2}' \quad (12)$$

Derivatives:

$$\frac{dL_1}{dt} = -k_1[L_1][I_1] + k_{-1}[L_1I_1] + k_6[L_1L_{1\text{-}1}'L_{1\text{-}2}'] - k_{-6}[L_1][L_{1\text{-}1}'][L_{1\text{-}2}']$$

$$\frac{dI_1}{dt} = -k_1[L_1][I_1] + k_{-1}[L_1I_1] + k_2[L_1I_1][L_1'] - k_{-2}[L_1L_1'][I_1] - k_3[T_1][T_2][I_1] + k_{-3}[T_1T_2I_1]$$

$$\frac{dL_1I_1}{dt} = k_1[L_1][I_1] - k_{-1}[L_1I_1] - k_2[L_1I_1][L_1'] + k_{-2}[L_1L_1'][I_1]$$

$$\frac{dL_1'}{dt} = -k_2[L_1I_1][L_1'] + k_{-2}[L_1L_1'][I_1]$$

$$\frac{dL_1L_1'}{dt} = k_2[L_1I_1][L_1'] - k_{-2}[L_1L_1'][I_1] - k_4[L_1L_1'][E] + k_{-4}[L_1L_1'E]$$

$$\frac{dT_1}{dt} = -k_3[T_1][T_2][I_1] + k_{-3}[T_1T_2I_1] - k_{11}[T_1][B_1] + k_{-11}[T_1B_1]$$

$$\frac{dT_2}{dt} = -k_3[T_1][T_2][I_1] + k_{-3}[T_1T_2I_1]$$

$$\frac{dT_1T_2I_1}{dt} = k_3[T_1][T_2][I_1] - k_{-3}[T_1T_2I_1]$$

$$\frac{dE}{dt} = -k_4[L_1L_1'][E] + k_{-4}[L_1L_1'E] + k_5[L_1L_1'E] - k_4[L_2L_2'][E] + k_{-4}[L_2L_2'E] + k_5[L_2L_2'E]$$

$$\frac{dL_1L_1'E}{dt} = k_4[L_1L_1'][E] - k_{-4}[L_1L_1'E] - k_5[L_1L_1'E]$$

$$\frac{dL_1L_{1-1}'L_{1-2}'}{dt} = k_5[L_1L_1'E] - k_6[L_1L_{1\text{-}1}'L_{1\text{-}2}'] + k_{-6}[L_1][L_{1\text{-}1}'][L_{1\text{-}2}']$$

$$\frac{dL_{1-1}'}{dt} = k_6[L_1L_{1\text{-}1}'L_{1\text{-}2}'] - k_{-6}[L_1][L_{1\text{-}1}'][L_{1\text{-}2}']$$

$$\frac{dL_{1-2}'}{dt} = k_6[L_1L_{1\text{-}1}'L_{1\text{-}2}'] - k_{-6}[L_1][L_{1\text{-}1}'][L_{1\text{-}2}']$$

$$\frac{dL_2}{dt} = -k_7[L_2][I_2] + k_{-7}[L_2I_2] + k_{10}[L_2L_{2-1}'L_{2-2}'] - k_{-10}[L_2][L_{2-1}'][L_{2-2}']$$

$$\frac{dI_2}{dt} = -k_7[L_2][I_2] + k_{-7}[L_2I_2] + k_8[L_2I_2][L_2'] - k_{-8}[L_2L_2'][I_2] - k_9[T_3][T_4][I_2] + k_{-9}[T_3T_4I_2]$$

$$\frac{dL_2I_2}{dt} = k_7[L_2][I_2] - k_{-7}[L_2I_2] - k_8[L_2I_2][L_2'] + k_{-8}[L_2L_2'][I_2]$$

$$\frac{dL_2'}{dt} = -k_8[L_2I_2][L_2'] + k_{-8}[L_2L_2'][I_2]$$

$$\frac{dL_2L_2'}{dt} = k_8[L_2I_2][L_2'] - k_{-8}[L_2L_2'][I_2] - k_4[L_2L_2'][E] + k_{-4}[L_2L_2'E]$$

$$\frac{dT_3}{dt} = -k_9[T_3][T_4][I_2] + k_{-9}[T_3T_4I_2]$$

$$\frac{dT_4}{dt} = -k_9[T_3][T_4][I_2] + k_{-9}[T_3T_4I_2]$$

$$\frac{dT_3T_4I_2}{dt} = k_9[T_3][T_4][I_2] - k_{-9}[T_3T_4I_2]$$

$$\frac{dL_2L_2'E}{dt} = k_4[L_2L_2'][E] - k_{-4}[L_2L_2'E] - k_5[L_2L_2'E]$$

$$\frac{dL_2L_{2-1}'L_{2-2}'}{dt} = k_5[L_2L_2'E] - k_{10}[L_2L_{2-1}'L_{2-2}'] + k_{-10}[L_2][L_{2-1}'][L_{2-2}']$$

$$\frac{dL_{2-1}'}{dt} = k_{10}[L_2L_{2-1}'L_{2-2}'] - k_{-10}[L_2][L_{2-1}'][L_{2-2}']$$

$$\frac{dL_{2-2}'}{dt} = k_{10}[L_2L_{2-1}'L_{2-2}'] - k_{-10}[L_2][L_{2-1}'][L_{2-2}']$$

$$\frac{dB_1}{dt} = -k_{11}[T_1][B_1] + k_{-11}[T_1B_1]$$

$$\frac{dT_1B_1}{dt} = k_{11}[T_1][B_1] - k_{-11}[T_1B_1]$$

Figure S12. Computational simulation of the Inhibitor B_1-gated dissipative system (Figure 4, State **B**). The kinetic scheme of the reactions associated with the time-dependent concentration changes during the dissipative transitions is summarized in above equations. Knowing the time-dependent concentration changes of the tetrahedron dimers T_1/T_2 and T_3/T_4 during the dissipative transitions, we computationally simulated the time-dependent concentration changes by using Matlab R2020a.

Kinetic equations of B_2 gated dissipative system (Figure 4, State C):

$$L_1 + I_1 \underset{k_{-1}}{\overset{k_1}{\rightleftharpoons}} L_1I_1 \quad (1)$$

$$L_1I_1 + L_1' \underset{k_{-2}}{\overset{k_2}{\rightleftharpoons}} L_1L_1' + I_1 \quad (2)$$

$$T_1 + T_2 + I_1 \underset{k_{-3}}{\overset{k_3}{\rightleftharpoons}} T_1T_2I_1 \quad (3)$$

$$L_1L_1' + E \underset{k_{-4}}{\overset{k_4}{\rightleftharpoons}} L_1L_1'E \quad (4)$$

$$L_1L_1'E \xrightarrow{k_5} E + L_1L_{1\text{-}1}'L_{1\text{-}2}' \quad (5)$$

$$L_1L_{1\text{-}1}'L_{1\text{-}2}' \underset{k_{-6}}{\overset{k_6}{\rightleftharpoons}} L_1 + L_{1\text{-}1}' + L_{1\text{-}2}' \quad (6)$$

$$L_2 + I_2 \underset{k_{-7}}{\overset{k_7}{\rightleftharpoons}} L_2I_2 \quad (7)$$

$$L_2I_2 + L_2' \underset{k_{-8}}{\overset{k_8}{\rightleftharpoons}} L_2L_2' + I_2 \quad (8)$$

$$T_3 + T_4 + I_2 \underset{k_{-9}}{\overset{k_9}{\rightleftharpoons}} T_3T_4I_2 \quad (9)$$

$$L_2L_2' + E \underset{k_{-4}}{\overset{k_4}{\rightleftharpoons}} L_2L_2'E \quad (10)$$

$$L_2L_2'E \xrightarrow{k_5} E + L_2L_{2\text{-}1}'L_{2\text{-}2}' \quad (11)$$

$$L_2L_{2\text{-}1}'L_{2\text{-}2}' \underset{k_{-10}}{\overset{k_{10}}{\rightleftharpoons}} L_2 + L_{2\text{-}1}' + L_{2\text{-}2}' \quad (12)$$

$$T_3 + B_2 \underset{k_{-12}}{\overset{k_{12}}{\rightleftharpoons}} T_3B_2 \quad (14)$$

Derivatives:

$$\frac{dL_1}{dt} = -k_1[L_1][I_1] + k_{-1}[L_1I_1] + k_6[L_1L_{1\text{-}1}'L_{1\text{-}2}'] - k_{-6}[L_1][L_{1\text{-}1}'][L_{1\text{-}2}']$$

$$\frac{dI_1}{dt} = -k_1[L_1][I_1] + k_{-1}[L_1I_1] + k_2[L_1I_1][L_1'] - k_{-2}[L_1L_1'][I_1] - k_3[T_1][T_2][I_1] + k_{-3}[T_1T_2I_1]$$

$$\frac{dL_1I_1}{dt} = k_1[L_1][I_1] - k_{-1}[L_1I_1] - k_2[L_1I_1][L_1'] + k_{-2}[L_1L_1'][I_1]$$

$$\frac{dL_1'}{dt} = -k_2[L_1I_1][L_1'] + k_{-2}[L_1L_1'][I_1]$$

$$\frac{dL_1L_1'}{dt} = k_2[L_1I_1][L_1'] - k_{-2}[L_1L_1'][I_1] - k_4[L_1L_1'][E] + k_{-4}[L_1L_1'E]$$

$$\frac{dT_1}{dt} = -k_3[T_1][T_2][I_1] + k_{-3}[T_1T_2I_1]$$

$$\frac{dT_2}{dt} = -k_3[T_1][T_2][I_1] + k_{-3}[T_1T_2I_1]$$

$$\frac{dT_1T_2I_1}{dt} = k_3[T_1][T_2][I_1] - k_{-3}[T_1T_2I_1]$$

$$\frac{dE}{dt} = -k_4[L_1L_1'][E] + k_{-4}[L_1L_1'E] + k_5[L_1L_1'E] - k_4[L_2L_2'][E] + k_{-4}[L_2L_2'E] + k_5[L_2L_2'E]$$

$$\frac{dL_1L_1'E}{dt} = k_4[L_1L_1'][E] - k_{-4}[L_1L_1'E] - k_5[L_1L_1'E]$$

$$\frac{dL_1L_{1-1}'L_{1-2}'}{dt} = k_5[L_1L_1'E] - k_6[L_1L_{1\text{-}1}'L_{1\text{-}2}'] + k_{-6}[L_1][L_{1\text{-}1}'][L_{1\text{-}2}']$$

$$\frac{dL_{1-1}'}{dt} = k_6[L_1L_{1\text{-}1}'L_{1\text{-}2}'] - k_{-6}[L_1][L_{1\text{-}1}'][L_{1\text{-}2}']$$

$$\frac{dL_{1-2}'}{dt} = k_6[L_1L_{1\text{-}1}'L_{1\text{-}2}'] - k_{-6}[L_1][L_{1\text{-}1}'][L_{1\text{-}2}']$$

$$\frac{dL_2}{dt} = -k_7[L_2][I_2] + k_{-7}[L_2I_2] + k_{10}[L_2L_{2\text{-}1}'L_{2\text{-}2}'] - k_{-10}[L_2][L_{2\text{-}1}'][L_{2\text{-}2}']$$

$$\frac{dI_2}{dt} = -k_7[L_2][I_2] + k_{-7}[L_2I_2] + k_8[L_2I_2][L_2'] - k_{-8}[L_2L_2'][I_2] - k_9[T_3][T_4][I_2] + k_{-9}[T_3T_4I_2]$$

$$\frac{dL_2I_2}{dt} = k_7[L_2][I_2] - k_{-7}[L_2I_2] - k_8[L_2I_2][L_2'] + k_{-8}[L_2L_2'][I_2]$$

$$\frac{dL_2'}{dt} = -k_8[L_2I_2][L_2'] + k_{-8}[L_2L_2'][I_2]$$

$$\frac{dL_2L_2'}{dt} = k_8[L_2I_2][L_2'] - k_{-8}[L_2L_2'][I_2] - k_4[L_2L_2'][E] + k_{-4}[L_2L_2'E]$$

$$\frac{dT_3}{dt} = -k_9[T_3][T_4][I_2] + k_{-9}[T_3T_4I_2] - k_{12}[T_3][B_2] + k_{-12}[T_3B_2]$$

$$\frac{dT_4}{dt} = -k_9[T_3][T_4][I_2] + k_{-9}[T_3T_4I_2]$$

$$\frac{dT_3T_4I_2}{dt} = k_9[T_3][T_4][I_2] - k_{-9}[T_3T_4I_2]$$

$$\frac{dL_2L_2'E}{dt} = k_4[L_2L_2'][E] - k_{-4}[L_2L_2'E] - k_5[L_2L_2'E]$$

$$\frac{dL_2L_{2-1}'L_{2-2}'}{dt} = k_5[L_2L_2'E] - k_{10}[L_2L_{2\text{-}1}'L_{2\text{-}2}'] + k_{-10}[L_2][L_{2\text{-}1}'][L_{2\text{-}2}']$$

$$\frac{dL_{2-1}'}{dt} = k_{10}[L_2L_{2\text{-}1}'L_{2\text{-}2}'] - k_{-10}[L_2][L_{2\text{-}1}'][L_{2\text{-}2}']$$

$$\frac{dL_{2-2}'}{dt} = k_{10}[L_2L_{2\text{-}1}'L_{2\text{-}2}'] - k_{-10}[L_2][L_{2\text{-}1}'][L_{2\text{-}2}']$$

$$\frac{dB_2}{dt} = -k_{12}[T_3][B_2] + k_{-12}[T_3B_2]$$

$$\frac{dT_3B_2}{dt} = k_{12}[T_3][B_2] - k_{-12}[T_3B_2]$$

Figure S13. Computational simulation of the Inhibitor B_2-gated dissipative system (Figure 4, State **C**). The kinetic scheme of the reactions associated with the time-dependent concentration changes during the dissipative transitions is summarized in above equations. Knowing the time-dependent concentration changes of the tetrahedron dimers T_1/T_2 and T_3/T_4 during the dissipative transitions, we computationally simulated the time-dependent concentration changes by using Matlab R2020a.

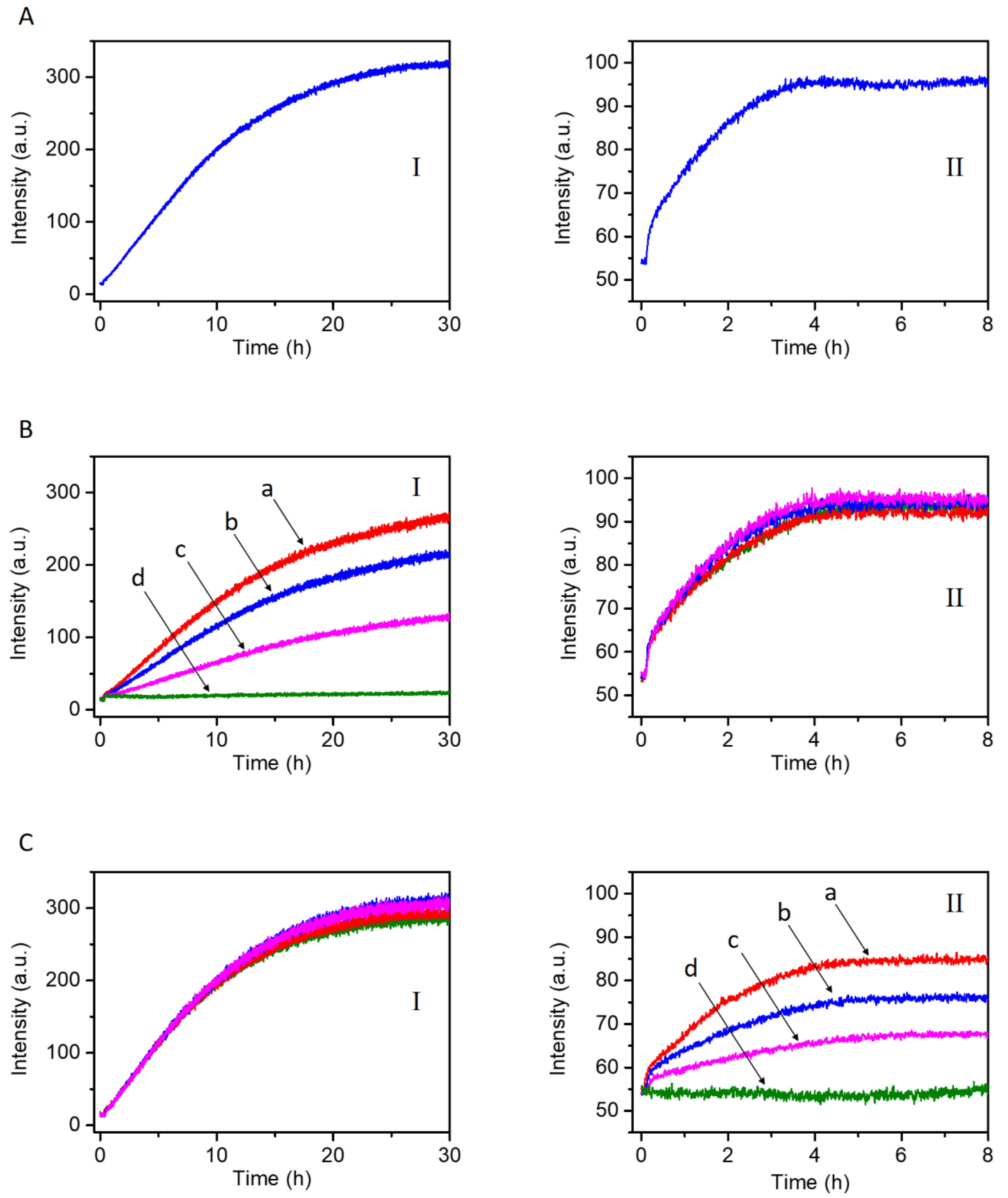

Figure S14. (A) Time-dependent Fluorescence intensity changes of the fragmented substrate of DNAzyme(**1**) and DNAzyme(**2**), in state **1**: Panel I- the Cy5-labled fragmented product of DNAzyme(**1**); Panel II- the FAM-labled fragmented product of DNAzyme(**2**); (B) Time-dependent fluorescence intensity changes of fragmented fluorophore-generated substrate cleaved by the DNAzymes in state **2**: Panel I- DNAzyme(**1**), in the presence of variable concentrations of B_1: (a) = 0.33 μM, (b) = 0.66 μM, (c) = 1 μM, (d) = 1.33 μM; Panel II-DNAzyme(**2**) in the presence of variable concentrations of B_1, (shown in Panel I), the DNAzyme(**2**) is unaffected by B_1. (C)

Time-dependent fluorescence intensity changes of the fragmented fluorophore-generated substrate cleaved by the DNAzymes in state **3**: Panel I-DNAzyme(**1**), in the presence of variable concentrations of B_2 outlined in Panel II, the DNAzyme(**1**) is unaffected by B_2; Panel II-DNAzyme(**2**) in the presence of different concentrations of B_2, (a) = 0.33 μM, (b) = 0.66 μM, (c) = 1 μM, (d) = 1.33 μM.

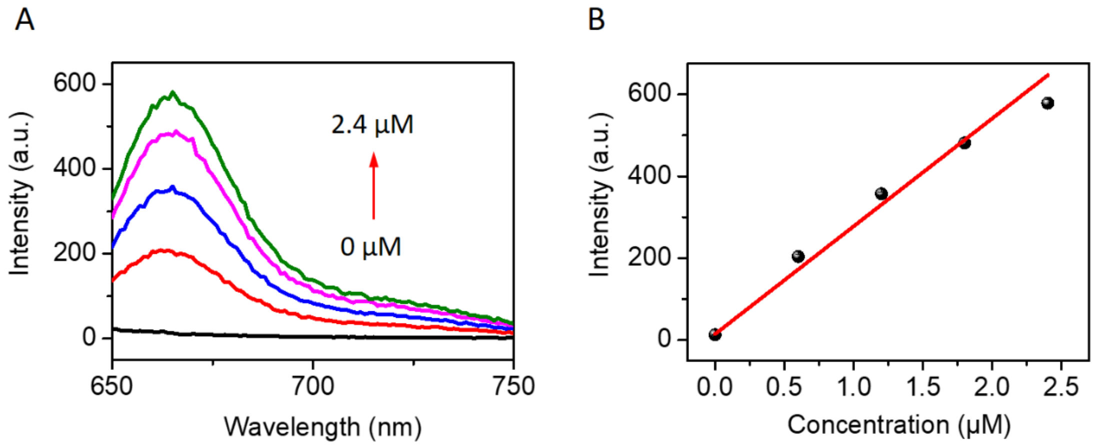

Figure S15. (A) Fluorescence spectra of the fluorophore Cy5-modified fragmented strand at different concentrations. (B) Calibration curve corresponding to the fluorescence intensities of Cy5-modified fragmented strand at variable concentrations (means ± standard deviation, n = 3).

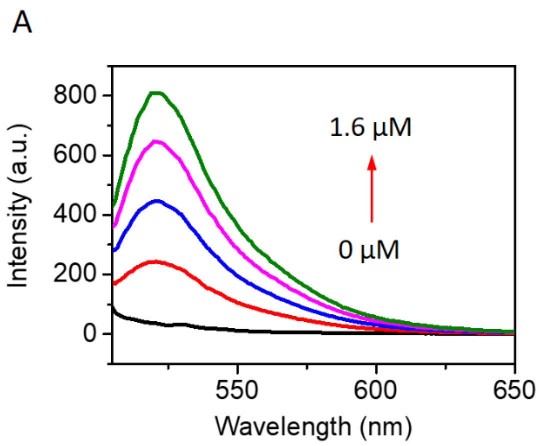

Figure S16. (A) Fluorescence spectra of the fluorophore FAM-modified fragmented strand at different concentrations. (B) Calibration curve corresponding to the fluorescence intensities of FAM-modified fragmented strand at variable concentrations (means ± standard deviation, n = 3).

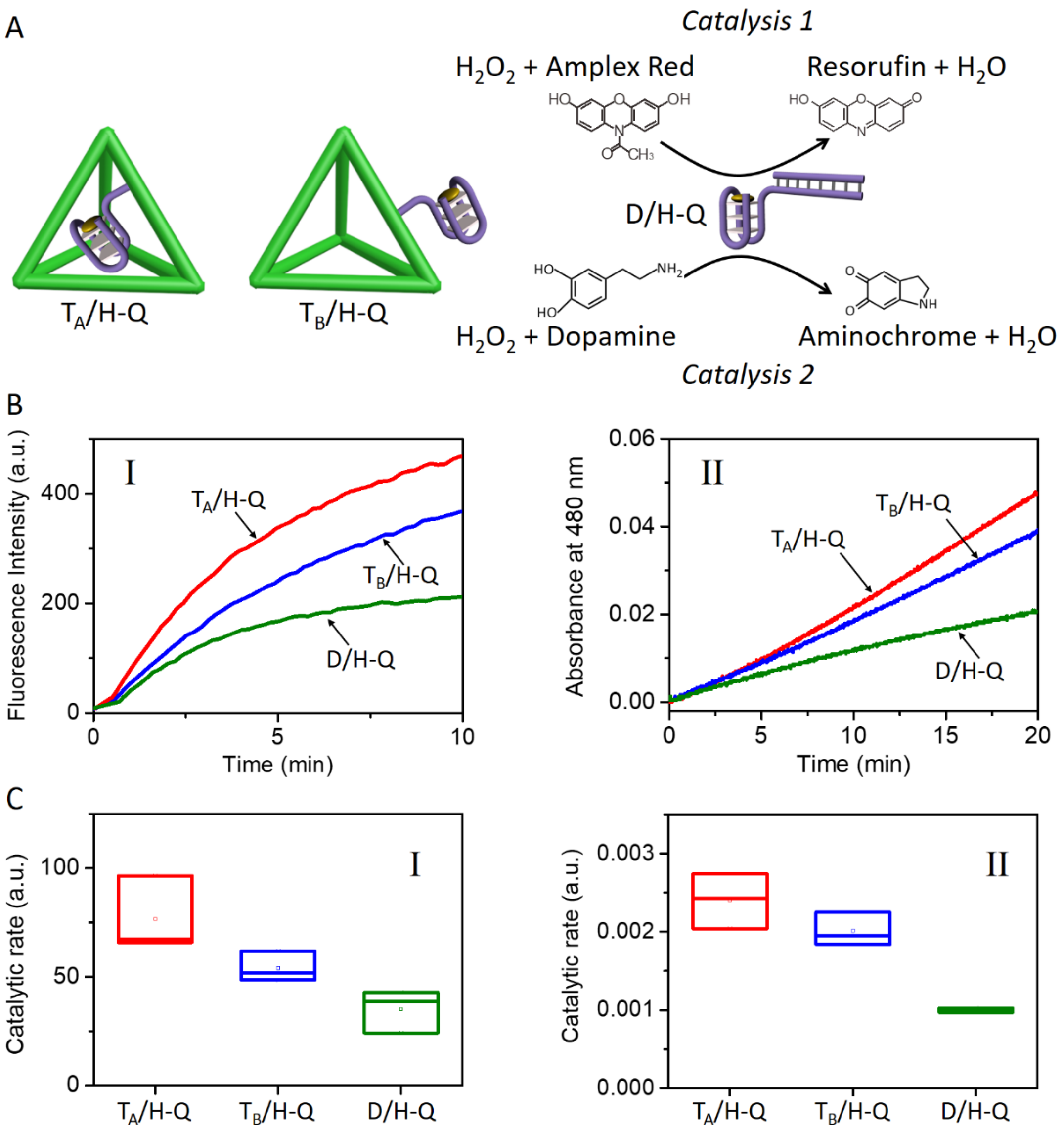

Figure S17. (A) Schematic configuration of the hemin/Q-quadruplex modified tetrahedra structures, T_A/H-Q and T_B/H-Q, and the analog hemin/G-quadruplex structure, D/H-Q. The detailed sequences of the respective structures are provided in the list of sequences, page s3. The catalytic features of the hemin/G-quadruplex units, H/Q, were evaluated by following the H_2O_2 catalyzed oxidation of Amplex Red to Resorufin or the H_2O_2 catalyzed oxidation of dopamine to aminochrome. (B) Panel I- Time-dependent fluorescence changes generated upon the H_2O_2 oxidation of Amplex Red (80 μM) to Resorufin (λ_{em} = 585 nm) by: TA/H-Q (0.1 μM), TB/H-Q (0.1 μM), D/H-Q (0.1 μM) in the presence of H_2O_2 (5 mM). Panel II- Time-dependent absorbance changes generated upon the H_2O_2 oxidation of dopamine (1 mM) to aminochrome (absorbance at 480 nm) by: TA/H-Q (0.2 μM), TB/H-Q (0.2 μM), D/H-Q (0.2 μM) in

the presence of H_2O_2 (5 mM). (C) Panel I-Catalytic rates corresponding to the oxidation of Amplex Red to Resorufin by the different catalytic systems. Error bars derived from N = 3 experiments. Panel II- Catalytic rates corresponding to the oxidation of dopamine to aminochrome by the different catalytic systems. Error bars derived from N = 3 experiments.

Table S1. Rate constants derived from the computational simulation of the dissipative system shown in Figure 1.

k_1	2.913 μM^{-1} min^{-1}	k_3	5 μM^{-2} min^{-1}	k_5	0.81 min^{-1}
k_{-1}	0.00001 min^{-1}	k_{-3}	0.0236 min^{-1}	k_6	0.028 min^{-1}
k_2	0.0845 μM^{-1} min^{-1}	k_4	5.00001 μM^{-1} min^{-1}	k_{-6}	0.00502 μM^{-2} min^{-1}
k_{-2}	50 μM^{-1} min^{-1}	k_{-4}	0.9988 min^{-1}		

Table S2. Rate constants derived from the computational simulation of the dissipative system shown in Figure 3.*

k_7	35 μM^{-1} min^{-1}	k_9	2.985 μM^{-2} min^{-1}	k_5	0.81 min^{-1}
k_{-7}	0.04 min^{-1}	k_{-9}	1.31 min^{-1}	k_{10}	20.001 min^{-1}
k_8	1.72 μM^{-1} min^{-1}	k_4	5.00001 μM^{-1} min^{-1}	k_{-10}	0.00001 μM^{-2} min^{-1}
k_{-8}	5.9 μM^{-1} min^{-1}	k_{-4}	0.9988 min^{-1}		

*For independent evaluation of the rate constants k_9 and k_{-9}, see Figure S10. The independently evaluated values of k_9, k_{-9} fit well with the simulated rate constants tabulated in Table S2, thus supporting the simulation process.

Table S3. Rate constants derived from the computational simulation of the gated dissipative system (Figure 4, State **A**).

k_1	2.913 μM^{-1} min^{-1}	k_{-4}	0.9988 min^{-1}	k_{-8}	5.9 μM^{-1}min^{-1}
k_{-1}	0.00001 min^{-1}	k_5	0.81 min^{-1}	k_9	2.985 μM^{-2} min^{-1}
k_2	0.0845 μM^{-1} min^{-1}	k_6	0.028 min^{-1}	k_{-9}	1.31 min^{-1}
k_{-2}	50 μM^{-1} min^{-1}	k_{-6}	0.00502 μM^{-2}min^{-1}	k_{10}	20.001 min^{-1}
k_3	5 μM^{-2} min^{-1}	k_7	35 μM^{-1} min^{-1}	k_{-10}	0.00001 μM^{-2} min^{-1}
k_{-3}	0.0236 min^{-1}	k_{-7}	0.04 min^{-1}		
k_4	5.00001 μM^{-1} min^{-1}	k_8	1.72 μM^{-1} min^{-1}		

Table S4. Rate constants derived from the computational simulation of the Blocker B_1-gated dissipative system (Figure 4, State **B**).

k_1	2.913 μM^{-1} min^{-1}	k_{-4}	0.9988 min^{-1}	k_{-8}	5.9 $\mu M^{-1}min^{-1}$
k_{-1}	0.00001 min^{-1}	k_5	0.81 min^{-1}	k_9	2.985 μM^{-2} min^{-1}
k_2	0.0845 μM^{-1} min^{-1}	k_6	0.028 min^{-1}	k_{-9}	1.31 min^{-1}
k_{-2}	50 μM^{-1} min^{-1}	k_{-6}	0.00502 $\mu M^{-2}min^{-1}$	k_{10}	20.001 min^{-1}
k_3	5 μM^{-2} min^{-1}	k_7	35 μM^{-1} min^{-1}	k_{-10}	0.00001 μM^{-2} min^{-1}
k_{-3}	0.0236 min^{-1}	k_{-7}	0.04 min^{-1}	k_{11}	30 $\mu M^{-1}min^{-1}$
k_4	5.00001 μM^{-1} min^{-1}	k_8	1.72 μM^{-1} min^{-1}	k_{-11}	0.00001 min^{-1}

Table S5. Rate constants derived from the computational simulation of the Blocker B_2-gated dissipative system (Figure 4, State **C**).

k_1	2.913 μM^{-1} min^{-1}	k_{-4}	0.9988 min^{-1}	k_{-8}	5.9 $\mu M^{-1} min^{-1}$
k_{-1}	0.00001 min^{-1}	k_5	0.81 min^{-1}	k_9	2.985 μM^{-2} min^{-1}
k_2	0.0845 μM^{-1} min^{-1}	k_6	0.028 min^{-1}	k_{-9}	1.31 min^{-1}
k_{-2}	50 μM^{-1} min^{-1}	k_{-6}	0.00502 $\mu M^{-2} min^{-1}$	k_{10}	20.001 min^{-1}
k_3	5 μM^{-2} min^{-1}	k_7	35 μM^{-1} min^{-1}	k_{-10}	0.00001 μM^{-2} min^{-1}
k_{-3}	0.0236 min^{-1}	k_{-7}	0.04 min^{-1}	k_{12}	28.5268 $\mu M^{-1} min^{-1}$
k_4	5.00001 μM^{-1} min^{-1}	k_8	1.72 μM^{-1} min^{-1}	k_{-12}	0.00001 min^{-1}
